# Correlation between oral microbiota and dry socket at different time periods on tooth extraction

**DOI:** 10.1080/20002297.2025.2485210

**Published:** 2025-04-04

**Authors:** Yujia Wu, Hujie Lyu, Xuliang Deng, Yong-Xin Liu, Ying He, Mingming Xu

**Affiliations:** a Department of Geriatric Dentistry, Peking University School and Hospital of Stomatology, NMPA Key Laboratory for Dental Materials, National Engineering Research Center of Oral Biomaterials and Digital Medical Devices, Beijing Laboratory of Biomedical Materials, Beijing Key Laboratory of Digital Stomatology, Beijing, P. R. China; b Genome Analysis Laboratory of the Ministry of Agriculture and Rural Affairs, Agricultural Genomics Institute at Shenzhen, Chinese Academy of Agricultural Sciences, Shenzhen, Guangdong, China; c Department of Life Sciences, Imperial College of London, London, UK

**Keywords:** Oral microbiota, dry socket, 16S rDNA sequencing, random forest, microbial network

## Abstract

**Background:**

Dry socket is a common post-extraction complication, characterized by the exposure of bone surfaces to the oral environment, leading to severe pain and potential infection. This study investigates the relationship between oral microbial composition and dry socket incidence in tooth extraction patients.

**Methods:**

From 87 patients (56 normal healing, 31 dry socket), 321 microbial samples were collected at pre-, med-, and post-extraction stages from saliva and the extraction sites, and all information was documented. All samples underwent 16S rDNA sequencing and amplicon analysis.

**Results:**

Dry socket patients exhibited distinct oral microbial diversity and composition. *Prevotella*, *Fusobacterium,* and *Haemophilus* strongly associated with the occurrence of dry socket. The microbial profiles in saliva revealed clearer temporal changes and healing/dry socket distinctions. The microbial network in the saliva of patients with dry socket exhibited key node/edge differences between med/post stages. Random forest analysis using pre-extraction saliva microbes to predict post-extraction symptoms, achieving a 75% accuracy rate in identifying the healthy group.

**Conclusion:**

*Haemophilus* and *Fusobacterium* were key microbes in dry socket development and prediction. Functional changes caused by alterations in microbial composition and structure might have been the reason for the different symptoms observed after tooth extraction.

## Introduction

In recent years, dry socket, a common painful complication after tooth extraction, has posed a challenge in clinical management in the field of oral medicine [1,2]. The main characteristic of dry socket is pain in the tooth socket, typically intensifying on the second or third day after extraction, often accompanied by delayed healing. After tooth extraction, healthy individuals recover quickly without severe pain, while patients who develop dry socket suffer severe pain due to direct exposure of bone and nerves to the oral environment. The most direct connection between different post-extraction manifestations is the variation in oral microbiota.

Research on microbiomes has grown in significance [3], and a growing number of studies have begun to focus on the role of oral microbiota in the development of oral diseases [4–6]. One study found that bacteria such as *Parvimonas*, *Peptostreptococcus* were over-represented in the oral microbiome of patients with dry socket, suggesting that these bacteria may play a key role in the pathogenesis of dry socket [7]. In addition, evidence suggested that bacterial biofilm formation may disrupt normal blood clot formation and stability in the tooth socket, increasing the risk of dry socket [8,9]. Most studies address the clinical management of dry socket, while its interaction with oral microbiota remains unexplored [10]. Therefore, this study aims to explore the differences in oral microbial composition between patients with dry socket and healthy patients and to investigate the dynamic changes in oral microbiota associated with the development and progression of this condition.

We particularly focus on identifying specific microbial communities associated with increased risk of dry socket. Through comparative analysis, we aim to identify potential biomarkers to provide new strategies for the prevention and treatment of dry socket. This study fills gaps in knowledge and provides evidence to guide clinicians, improving both care quality and patient outcomes.

## Materials and methods

### Experimental design and sample collection

This is a prospective observational study aimed at systematically observing and recording the incidence of dry socket in patients undergoing the extraction of the third molar and the changes in the oral microbiome during this process. The study was approved by the Ethics Committee of Peking University School and the Hospital of Stomatology (Approval Number: PKUSSIRB - 20086), and written informed consent was obtained from all participants. The study included subjects scheduled for the extraction of third molars at the Geriatric Dentistry Department of Peking University Hospital of Stomatology. Subjects eligible for the study had to meet specific criteria, including an age between 18 and 50 years, good general health and the absence of any systemic diseases. Subjects who met any of the following exclusion criteria were excluded from the study: a history of coagulopathy or known bleeding disorders to minimize complications during and after surgical procedures; pregnant or breastfeeding women due to the potential effects of the study procedures and radiation exposure; patients currently taking medications known to affect wound healing (e.g. immunosuppressants and steroids) to control for confounding variables; individuals who had experienced pericoronitis of the wisdom tooth in the past month; and individuals who had used antibiotics in the past month.

Patient information and dry socket identification: Comprehensive demographic information was collected from each participant at the beginning of the study, including details of their age, gender, and relevant aspects of their medical history. In addition to demographic information, a questionnaire was also administered to all participants to assess their oral hygiene habits and to record their smoking status and medication use. The extraction of the third molars was performed by an oral and maxillofacial surgeon with 10 years of clinical experience, and detailed information regarding the specific techniques of third molar extraction was recorded post-surgery. Patients were required to attend follow-up visits on the 3rd to 5th day and the 6th to 10th day after tooth extraction. During each follow-up visit, a thorough examination of the oral condition was conducted, and the healing of the extraction site and facial swelling were recorded. Post-operative pain was assessed using a Visual Analogue Scale (VAS), where participants were asked to score their pain level on a scale from 0 to 10, providing valuable insights into the intensity of pain experienced post-operatively. The identification of dry socket was conducted using standardized criteria established *a* *priori,* including clinical signs and symptoms such as severe pain and delayed wound healing.

Sample Collection: The methodology for saliva collection is based on the established protocol from the US Human Microbiome Project [11]. A total of 1 ml of spontaneously secreted, non-stimulated saliva was collected. For the collection of periodontal subgingival plaque and microbiota within the extraction socket, an adsorption technique was employed using 2 mm *10 mm filter paper strips (Whatman, UK). These strips are handled with sterile instruments, undergo high-pressure steam sterilization prior to use, and are subsequently dried. A filter paper strip is inserted into the periodontal pocket along the tooth’s surface, and pressure is applied for 30 s until resistance is met. The strip is then transferred into a 0.6 mL sterile centrifuge tube. Post-sampling, the samples are promptly placed in a temperature-controlled icebox to ensure preservation and transportation. First, on the day of tooth extraction (day 0), 1 ml of saliva was collected from each patient, and the filter paper strips were inserted into the third molar periodontal pockets to collect plaque. All patients were required to attend follow-up visits on the 3rd and 7th days after extraction, where they were registered and saliva and microbiota from the extraction site were collected. The patients on the day of extraction were labeled as the pre stage, those from the 3rd to 5th day as the med stage and those from the 6th to 10th day as the post stage. In the intermediate follow-up, patients were divided into healthy and dry socket groups based on their specific symptoms. After diagnosis, patients with dry socket received intervention treatment, which involved repeated irrigation of the extraction site with hydrogen peroxide and normal saline under local anesthesia until all necrotic tissue was completely cleared from the socket, followed by the placement of iodoform gauze to pack the socket. According to the sampling site, it can be divided into saliva and extraction socket groups. The specific grouping can refer to Supplementary Table S1.

Grouping information: In total, we enrolled 400 patients, including extraction of 476 teeth. Finally, we included 102 subjects, among which 31 patients diagnosed with dry socket (D), 56 exhibited normal healing and were classified as healthy (H). The remaining 15 patients showed impaired wound healing (I), which did not form a describable or homogeneous group and were therefore excluded from further analysis. Consequently, the final analysis included 87 patients (31 with dry socket and 56 with normal healing) and 321 samples met the quality standards. The sampling symptoms were dry socket (D) and healthy (H). The sampling sites were saliva (S) and extraction sockets (E) (samples were taken from the periodontal pocket (P) before tooth extraction). The sampling times were divided into three periods: pre, med and post. The pre stage refers to the time before the surgical operation on the day of tooth extraction, the med stage refers to the 3rd day after tooth extraction and the post stage refers to the 7th day after tooth extraction. All samples could be categorized into 12 different groups based on two different sampling locations: saliva (S) and extraction socket/periodontal pocket (E/P); two different symptoms: dry socket (D) and health (H); and three different sampling times: pre, med, post. The groups are as follows: HSpre, HSmed, HSpost, HPpre, HEmed, HEpost, DSpre, DSmed, DSpost, DPpre, DEmed, DEpost. The specific meanings of the letters can be found in Supplementary Table S1.

### DNA extraction, PCR amplification and sequencing

Total genomic DNA from samples was extracted using sodium dodecyl sulfate (SDS) method. DNA concentration and purity were monitored on 1% agarose gels. According to the concentration, DNA was diluted to 1ng/μL using sterile water. 16S rDNA genes of distinct regions 16S V3-V4 were amplified using specific primer 16S V4: 515F-GTGCCAGCMGCCGCGGTAA & 806 R-GGACTACHVGGGTWTCTAAT with the barcode. All PCR reactions were carried out with Phusion® High-Fidelity PCR Master Mix (New England Biolabs). Mix the same volume of 1X loading buffer (containing SYBR green) with PCR products and operate electrophoresis on 2% agarose gel for detection. Samples with bright main strip between 400-450 bp (16S) were chosen for further experiments. The selected PCR products were mixed in equidensity ratios and purified with Qiagen Gel Extraction Kit (Qiagen, Germany). Sequencing libraries were generated with the TruSeq® DNA PCR-Free Sample Preparation Kit (Illumina, USA) following manufacturer’s recommendations and index codes were added. The library quality was assessed on the Qubit® 2.0 Fluorometer (Thermo Scientific) and Agilent Bioanalyzer 2100 system. Finally, the library was sequenced on an Illumina NovaSeq 6000 platform, and 250 bp paired-end reads were generated.

### Bioinformatics analysis on 16S rDNA gene profiling

The primary data analysis was conducted using the EasyAmplicon pipeline [12]. In this study, 386 post-sequencing samples from all groups (discussed in the experiment design section) were screened, with each sample receiving approximately 50,000 reads. This dataset has been uploaded to the Genome Sequence Archive in the National Genomics Data Center, and the project number is PRJCA9613.

To ensure the accuracy of subsequent data analysis, the 16S rDNA gene sequences were processed using USEARCH [13,14], VSEARCH [15] and user-defined scripts. Metadata were provided in Supplementary Table S1b. Specifically, the reads were processed mainly by VSEARCH with the following steps: joining paired-end reads and relabelling sequences (fastq_mergepairs); removing barcodes, primers and filtering low-quality reads (-fastx_filter); and finding unique reads (-fastx_uniques). Unique sequences were denoised into ASV (−unoise3) (using USEARCH). ASVs were aligned with the Ribosomal Database Project (RDP) database to remove chimeras (-uchime_ref) and sequences from host organisms [16]. The ASV table was generated by VSEARCH (-usearch_global). The taxonomy annotation was generated by -sintax and used the script (-otutab_filter_nonBac.R) to remove non-bacterial contaminants. Alpha-diversity, beta-diversity and microbial composition analysis were performed using the ‘Taxonomic Diversity’ and ‘Composition Analysis’ scripts from EasyAmplicon pipeline [12,17]. Differential ASV abundance and taxonomic analyses were conducted using the Wilcoxon rank-sum test based on ASVs with a relative abundance of more than 0.2% for each group. The corresponding *P*-values were adjusted for multiple testing correction using the False Discovery Rate (FDR) set at 0.2. The ASV sequences and table were input into PICRUSt2 and BUGBASE (Greengenes database results were used for BUGBASE functional annotation) software for functional and phenotype prediction [18,19]. Functional annotations of prokaryotic taxa were carried out using FAPROTAX [20] from ImageGP 2 [21]. The representative sequences were used to construct phylogenetic trees and perform taxonomic annotation and were imported into STAMP [22] for biomarker identification.

Additionally, we used the ggClusterNet package [23] to predict the microbial interaction networks and employed the randomForest package [24] to identify markers associated with dry socket occurrence. The ggClusterNet was used to construct co-occurrence networks after removing ASVs with a relative abundance of less than 0.01%. Robust Pearson correlation scores (|r| > 0.9) and statistically significant correlations were tested (*P* < 0.05). Topological features, including degree, betweenness and closeness centrality, were measured at the node level, and the statistical differences between networks were compared using the Wilcoxon rank-sum test. Based on topological features, the role of ASVs within networks was measured by their within-module connectivity (Zi) and participation index (Pi) [25]. The Meconetcomp was used to compare between-network edges and nodes and microbial dissimilarities between different groups [26]. We analyzed the relative abundance of bacterial taxa in oral microbiota across different taxonomic levels, including phylum, class, order, family and genus, using the randomForest package in R with default parameters. For random forest analysis, a subset of samples was first selected as the training set to train the data, and another subset was used as the test set to assess whether the trained model could predict the results of the test set. We randomly selected 50% of the samples as the training set and 50% as the test set. Predictive classification models were generated using the randomForest function (importance = TRUE, proximity = TRUE), while the rfcv() function was employed for cross-validation to identify relevant features. Finally, we visualized feature importance and cross-validation curves with the ggplot2 package [27] in R software.

## Results

### Distinct oral microbial diversity in dry socket patients compared to healthy individuals

**Figure 1.**
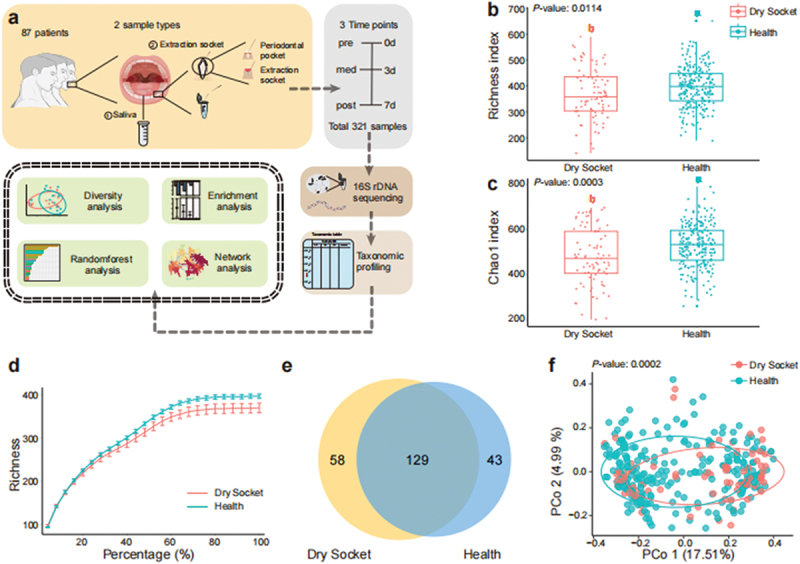
Oral microbial diversity in dry socket differs from health group. (a) Overview of sample collection, experimental design and analysis result. (b/c) alpha diversity of oral microbiota in the dry socket and healthy groups was compared using various indices, such as richness index (b) and Chao1 index (c), and visualized through boxplots. In the boxplot, the middle line represents the median, and the upper and lower edges represent the 75th and 25th percentiles, respectively. The whiskers extend to 1.5 times the interquartile range from the edges of the box. (d) Rarefaction curve of richness in the dry socket and healthy groups. (e) Venn diagram showing the overlap of enriched microbes between the dry socket and health group. (f) Principal coordinates analysis (PCoA) with Bray-Curtis distances, demonstrating the separation of oral microbiota between the two groups along the first coordinate axis (*p* < 0.05, Adonis by permutational multivariate analysis of variance).

Three hundred and twenty-one samples were collected from 87 patients and had two sample sites with three sampling times (Figure 1a and details in the Methods). Initially, we grouped all samples by dry socket and healthy symptoms, ignoring sites and time, to compare microbial diversity. Comparison of various alpha diversity indices revealed significant differences between the dry socket and healthy group, the mean richness index 369.615 in dry socket and 396.703 in health indicated that the difference in the mean values between the dry socket and healthy groups was 27.088 and an adjusted *P*-value of 0.0114 (Figure 1b). For the Chao1 index, the mean difference between groups was 48.801, with an adjusted *P*-value of 0.0003 (Figure 1c). The rarefaction curve showed that, after stabilization, microbial abundance in the dry socket group was lower (Figure 1d). A Venn diagram illustrated 58 unique amplicon sequence variants (ASVs) in the dry socket group, 43 in the healthy group and 129 shared between both groups (Figure 1e). Beta diversity analysis confirmed significant differences in microbial diversity between the two groups with *P*-value 0.0002 (Figure 1f). In addition, oral microbiota diversity was analyzed by sampling time and locations (Supplementary Figure S1 & Supplementary Table S2). Significant differences were observed between saliva and extraction sites, but no differences were found between periodontal pockets and extraction sockets (Supplementary Figure S1a). Beta diversity varied significantly between the pre and post stages as well as between the pre and med stages (Supplementary Figure S1b & c). Overall, the oral microbiota diversity in patients with dry socket was distinct from that of the healthy group.

### Oral microbiota of patients with dry socket differs from healthy in composition and functions

**Figure 2.**
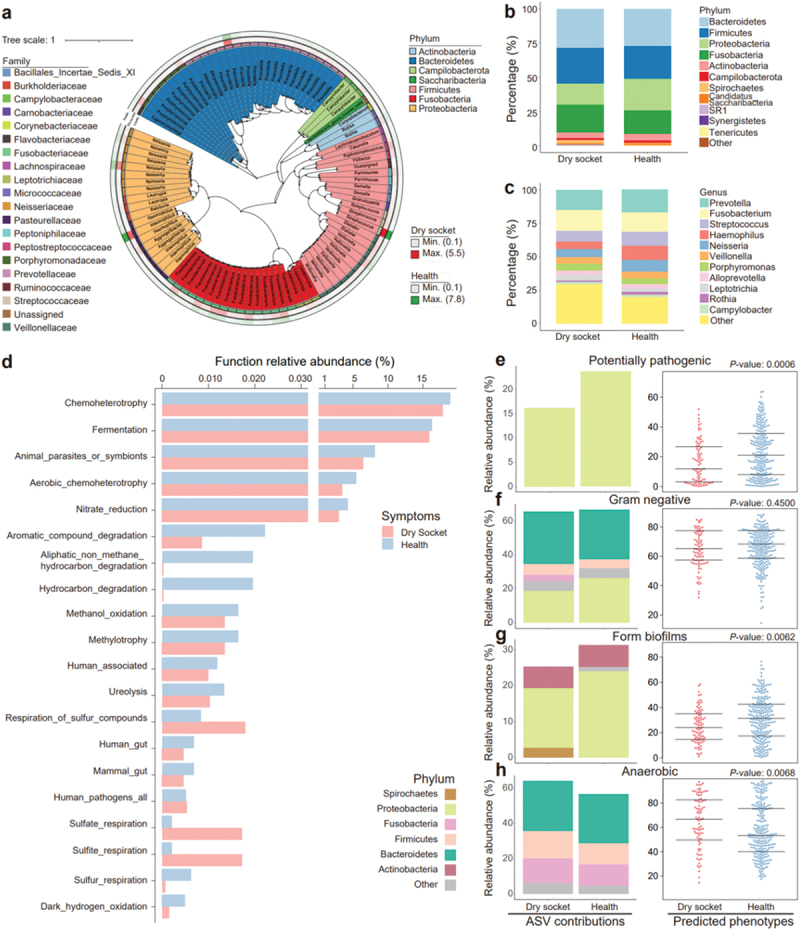
Microbial composition and functions of oral microbiota in the dry socket group differ from the healthy group. (a) Phylogenetic tree of microbiota in the dry socket and health groups, showing evolutionary relationships between groups. The inner background colored in phylum-level, the outer ring colored in family-level and outmost two heatmaps show the relative abundance in percentage of microbes in dry socket and healthy group. The tree scale represents evolutionary distance. (b/c) stacked bar chart showing the differences in oral microbiota composition at the phylum (b) and genus (c) levels between the dry socket and healthy groups. (d) FAPROTAX functional annotation of microbiota in the dry socket and healthy groups. The x-axis represents relative abundance of microbial function, with different colors indicating different groups. (e–h) BUGBASE functional annotation of oral microbiota in the dry socket and healthy groups. We used potentially pathogenic (e), Gram-negative (f), form biofilms (g) and anaerobic (h) as examples. The y-axis shows relative abundance, x-axis shows symptoms and the bottom labels indicate the analyzed types. The number of sample size: dry socket (*n* = 31), health (*n* = 56).

The phylogenetic tree displayed the evolutionary relationships among ASVs. It showed that oral microbes were mainly enriched in four phyla: Bacteroidetes, Firmicutes, Fusobacteria and Proteobacteria (Figure 2a). A stacked bar chart revealed enrichment of Proteobacteria in the healthy group and Fusobacteria were more enriched in the dry socket group (Figure 2b). At the genus level, *Haemophilus* and *Neisseria* were relatively depleted in the dry socket group (Figure 2c).

We utilized FAPROTAX for preliminary functional annotation and observed that functions such as chemoheterotrophy, aerobic chemoheterotrophy and nitrate reduction were dominant in the oral microbiota (Figure 2d, Supplementary Figure S2 & Supplementary Table S3). Notably, these functions were less enriched in the dry socket group compared to the healthy group. In contrast, the healthy group demonstrated higher enrichment of aromatic compound degradation, aliphatic non-methane hydrocarbon degradation and hydrocarbon degradation functions (Figure 2d). The enrichment of sulfur compound functions was relatively higher in the dry socket group (Figure 2d, Supplementary Figure S2 & Supplementary Table S3). BUGBASE annotation showed that potentially pathogenic-related function was less prevalent in the dry socket group (*p-*value 0.0006) and primarily linked to Proteobacteria (Figure 2e). The relative abundance of Gram-negative bacteria between dry socket and healthy groups was similar across groups (*P*-value 0.4500), and the proportion of Proteobacteria was relatively lower, while Fusobacteria was higher in the dry socket group (Figure 2f). Form biofilms-related functions differed between the groups (*P*-value 0.0062), and the relative abundance of Proteobacteria was found to be higher in the healthy group (Figure 2g). The dry socket group showed a greater contribution of Firmicutes to anaerobic-related functions compared to the healthy group (Figure 2h & Supplementary Table S4).

### Salivary microbial composition and functional change in dry socket and healthy patients at different periods of tooth extraction

**Figure 3.**
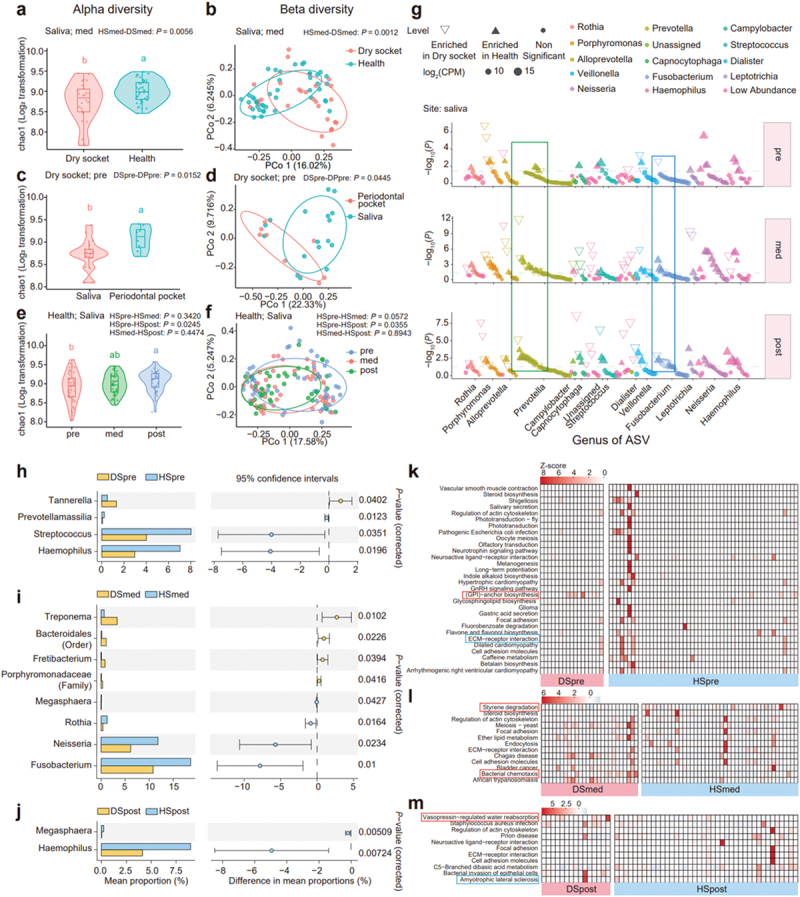
Salivary microbial composition and functions change with time in dry socket group. (a/b) alpha and beta diversity of oral microbiota in dry socket and healthy samples from the saliva sampling site at the med stage. (c/d) diversity across saliva and periodontal pocket site in dry socket patients during the pre-medical stage. (e/f) diversity at the saliva site in healthy samples at pre, med, post time. (g) Manhattan plot showing the differences in oral microbiota at the saliva site between dry socket and healthy individuals across pre, med, post stage. Each dot or triangle represents an ASV. Hollow or solid triangles indicate ASVs enriched in the dry socket or health groups, respectively (adjusted *p* < 0.05, Wilcoxon rank-sum test). ASVs are colored by genus level. CPM, counts per million. (h) Comparison of key differential microbes in saliva between the dry socket and healthy groups during different sampling time. *p* < 0.05, data within 95% confidence intervals. Blue bars represent healthy saliva, yellow bars represent dry socket saliva, with bar extensions showing group differences. The y-axis lists genus-level taxa, which are annotated at the highest identifiable level if genus is unavailable. (i) Heatmaps showing enriched functions in saliva microbiota based on PICRUSt2 during the pre, med and post stages in the dry socket and healthy groups. Different functions corresponding to groups are shown below in each plot, with each column representing an individual sample. Note: D represents the dry socket group, H represents the healthy group. S stands for saliva, E for extraction site, P for periodontal pocket. Pre indicates pre-extraction, med indicates after 3 days of medical treatment teeth extraction and post indicates after 7 days of teeth extraction.

We compared the microbial diversity differences among various groups under specific classifications (Supplementary Figure S3a-c). Significant microbial diversity differences were observed between saliva samples from dry socket and healthy groups at the med stage (alpha diversity with *P*-value 0.0056, beta diversity with *P*-value 0.0012) (Figure 3a, b). While the dry socket group displayed distinct diversity patterns across the different sampling sites at the pre stage (alpha diversity with *P*-value 0.0152, beta diversity with *P*-value 0.0445) (Figure 3c, d). When analyzing the diversity of different groups at various time points of tooth extraction, only the healthy group showed differences in saliva samples at pre and post extraction period (alpha diversity with *P*-value 0.0245, beta diversity with *P*-value 0.0355) (Figure 3e, f).

To explore the changes in oral microbiota over different sampling periods, a comparative analysis of the microbiota in the saliva of patients in health and dry socket at different period after tooth extraction was visualized using Manhattan plots. *Prevotella* exhibited low enrichment in the pre stage but became more enriched in the med and post stages. It was particularly enriched in the dry socket group during the med stage (Figure 3g, green box). *Fusobacterium* was more enriched in the healthy group at the med stage, and the dry socket group had higher levels at the post stage (Figure 3g, blue box). Subsequently, we compared and identified the microbes at saliva site that showed significant differences between the dry socket and healthy groups at pre, med, post time. Before the tooth extraction, only *Tannerella* was more abundant in the dry socket group (mean proportion 2.198 %, *P*-value 0.0402) (Figure 3h). During the med stage, the dry socket group had higher levels of *Treponema* (mean proportion 3.330 %, *P*-value 0.0102) and *Fretibacterium* (mean proportion 0.809 %, *P*-value 0.0394), while the healthy group had more *Neisseria*, and *Rothia* (mean proportion 11.846 % and 1.260 %, *P*-value 0.0234 and 0.0164) (Figure 3i). At the post stage, *Haemophilus* (mean proportion 8.961%, *P*-value 0.0072) were more abundant in healthy group (Figure 3j).

Next, we annotated and compared the functions of saliva microbiota between the dry socket and healthy groups at different time points using PICRUSt2 and ggpicrust2[28]. In the pre stage, the highest enrichment function in dry socket group was Glycosylphosphatidylinositol(GPI)-anchor biosynthesis. Conversely, the healthy group exhibited significant enrichment in functions related to ECM−receptor interaction (Figure 3k & Supplementary Figure 4a). During the med stage, the dry socket group showed enrichment in functions related to Bacterial chemotaxis and Styrene degradation (Figure 3l & Supplementary Figure 4b). In the post stage, the dry socket group demonstrated primary enrichment in Vasopressin−regulated water reabsorption, while the healthy group exhibited enrichment in functions linked to Amyotrophic lateral sclerosis (Figure 3m & Supplementary Figure 4c).

## Tooth extraction surgery and postoperative interventions for dry socket influence the oral microbial network

**Figure 4.**
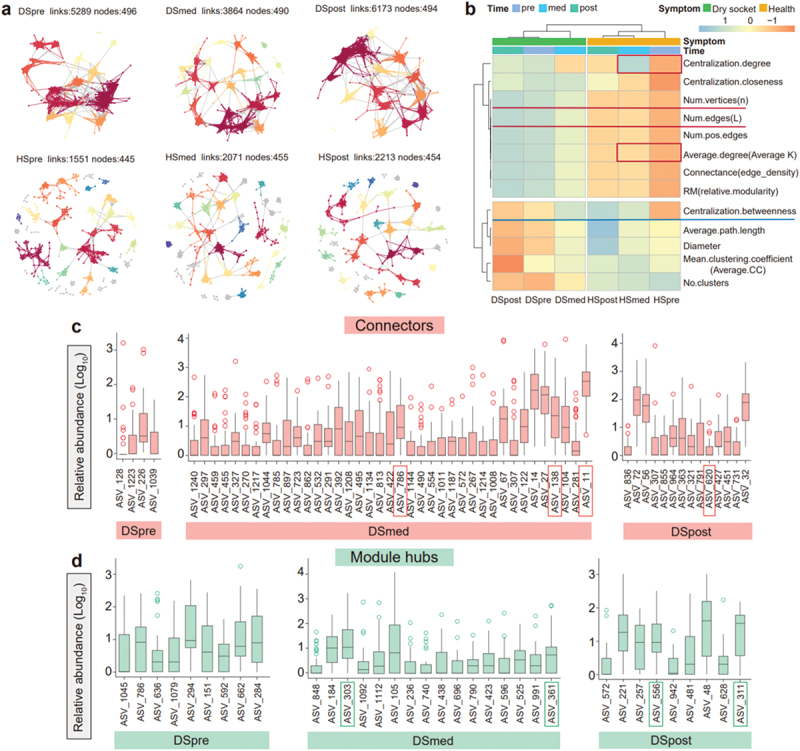
Salivary microbial network changes over time in dry socket and healthy groups. (a) Microbial networks shows variations in salivary microbiota between dry socket patients and healthy individuals across different time points. (b) Heatmap comparison of salivary microbial network properties between the health and dry socket groups across different time stages. (c/d) box plot showing microbial differences at key connectors (c) and module hubs (d) in dry socket patients’ saliva across three tooth extraction periods. Analysis based on within-module connectivity (Zi) and among-module connectivity (pi), nodes can be classified into four types: module hubs, connectors, network hubs and peripherals.

We analyzed the microbial networks in saliva across three time points in dry socket patients and performed similar analysis for the healthy group. In pre stage, the saliva microbial network of the dry socket group was higher connection than the healthy group (links of DSpre were 5289 and HSpre were 1551, nodes of DSpre were 496 and HSpre were 445) (Figure 4a). The dry socket group exhibited a greater number of edges, which decreased during the med stage (from 5289 to 3864) (Figure 4a). The microbial network of the healthy group showed relatively stable nodes and links before and after tooth extraction (with links around 2000 and nodes around 450) (Figure 4a). Based on the network metrics, we found that dry socket patients and healthy patients could be classified into distinct clusters (Figure 4b). The network in the healthy group was dominated by a few key nodes and edges (Num.vertices (n) and Num.edges (L)) compared to the dry socket group (Figure 4b, highlighted with a red underline). During the med time, the dry socket group showed reduced Centralization.degree and Average.degree level which decreased connectivity and communication efficiency between microbes (Figure 4b, red box). Furthermore, an increase in Centralization.betweenness underscored the role of specific microbes as potential biomarkers influencing dry socket development (Figure 4b, a blue underline). Interventions during the med stage appeared to rapidly restore the microbial network. Notably, the dry socket group’s network was larger and more centralized.

Using the ZiPi method, we identified key microbial nodes (details were showed in Supplementary Table S5). We found that in the saliva of the dry socket group during the med-extraction period, connectors were predominantly from the Bacteroidetes, with genera such as *Alloprevotella* (ASV_138) and *Prevotella* (ASV_786), and the relative abundance of *Fusobacterium* (ASV_11 & 14) was also higher (Figure 4c). Module hubs during the med stage of the dry socket group were enriched in Firmicutes and Bacteroidetes, with *Prevotella* (ASV_361), *Fretibacterium* (ASV_303) serving as core functional microbes (Figure 4d). In the post-extraction period, key microbes in the dry socket saliva microbial network decreased, with connector microbes like the *Prevotella* (ASV_620) reducing (Figure 4c). *Haemophilus* (ASV_311) and *Fusobacterium* (ASV_556) as module hub microbes were more abundant (Figure 4d).

## Random forest predicts dry socket

**Figure 5.**
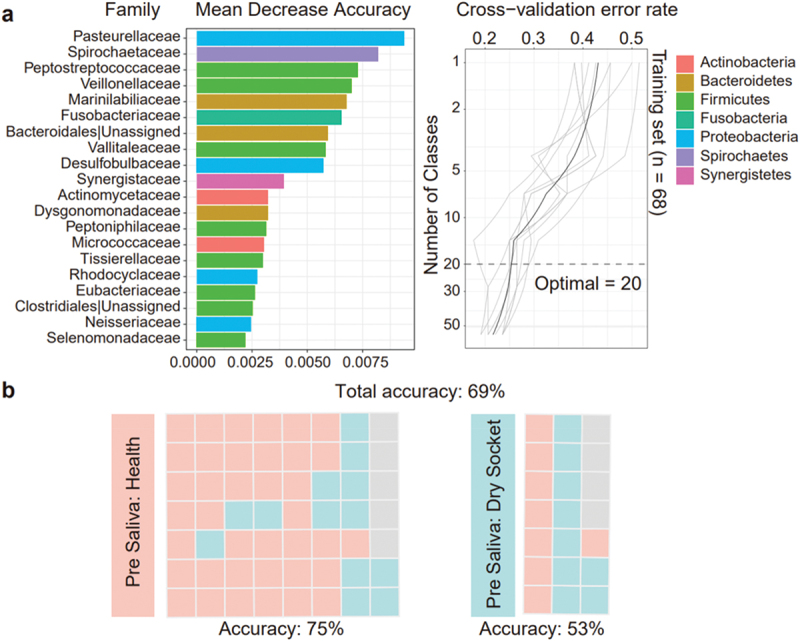
Random forest prediction of dry socket occurrence. (a) The top 20 bacterial families were identified using random forest classification of oral salivary microbiota abundance in health and dry socket groups at the pre stage. Biomarker taxa are ranked by their importance to model accuracy. The inset shows the 10-fold cross-validation error based on the number of input families used to differentiate health and dry socket microbiota. (b) Predictions for dry socket vs. health in pre-extraction salivary samples: red blocks indicate health predictions, blue blocks indicate dry socket predictions.

To predict the likelihood of dry socket occurrence after tooth extraction, we utilized oral microbes before tooth extraction. We compared the effects of different taxonomic levels on prediction accuracy and found that the family level had the highest accuracy (Supplementary Figure S5a & b). It was observed that the cross-validation error curve tended to be stable when 20 families were selected and as biomarker taxa. Among these, families such as Pasteurellaceae to Desulfobulbaceae displayed a mean decrease in accuracy ranging from 0.0094 to 0.0057 (Figure 5a), highlighting their significant contribution to the model’s predictive power. For dry socket prediction, we found the pre-extraction prediction accuracy for the healthy group was 75%, while the dry socket group exhibited a lower correct prediction rate of 53% (Figure 5b).

## Discussion

In this study, we demonstrated that oral microbiota diversity, microbial composition and functionality differed between patients with dry socket and healthy individuals. The microbial diversity of individuals who subsequently developed dry socket differed from that of the healthy group. Notably, variations in microbial diversity were observed at different sampling times and sites, suggesting a close association between oral microbes and the development of dry socket. Further analysis of the composition and functionality of oral microorganisms confirmed that the dry socket exhibited characteristics consistent with the results of the diversity analysis. Functionally, we observed a significant reduction in chemoheterotrophic and nitrate reduction functions in the dry socket group, while functions related to sulfur-related transformation were enriched, which may be associated with halitosis [29,30]. These results indicated that the development of dry socket affected the metabolic functions of the oral microbes, reducing their ability to decompose food residues and shed epithelial cells, thereby weakening the efficiency of the nutrient cycle [31–34]. Additionally, the reduction of parasitic and symbiotic microbes within the dry socket group resulted in decreased diversity and abundance of oral microbiota, rendering the oral cavity more susceptible to external pathogens [35,36]. Conversely, the healthy group showed particularly pronounced functions in the degradation of aromatic compounds and hydrocarbons, which are crucial for breaking down harmful compounds and reducing potential irritation or toxicity to healing tissues. Hence, protecting oral health and promotes the stability of the ecological environment [37]. These findings underscore the pivotal role of oral microbiota in the occurrence of dry socket and highlight how microbial differences influence various post-extraction symptoms, with specific microbial functions closely associated with the different manifestations that occurred after tooth extraction.

The timing of sampling also significantly impacted oral microbiota composition and function in dry socket patients and healthy individuals. Comparative analyses at the genus level of microbiota and their associated functions in saliva samples across tooth extraction periods revealed important insights. For instance, *Porphyromonas*, a genus associated with periodontal disease [38], was more abundant in the dry socket group during the pre-extraction period. During the med stage, dry socket patients exhibited a relative enrichment of *Treponema* and *Fretibacterium*, which are primarily associated with the periodontitis, dental plaque formation and oral tissue destruction [39,40]. *Fusobacterium* and *Neisseria* were enriched in the healthy group, and studies have also highlighted their anti-inflammatory and oral protective effects [41,42]. However, some studies demonstrated that *Fusobacterium nucleatum* played a role in the development of periodontal disease [43]. Overall, the pathogenesis of dry socket involves a dynamic balance of microbiota. In the post period, the primary microbes were *Megasphaera* and *Haemophilus*, with enriched functions related to neural protection and tissue repair. It was not difficult to notice the dynamic shifts in oral microbial composition and function between the dry socket and healthy groups over time. The observed microbial changes may induce the varying symptoms observed across different time stages, emphasizing the need for further investigation into their functional role.

For the predicted microbial functions, we observed notable temporal variations between the dry socket and healthy groups in saliva samples. At the pre stage of sampling, the glycosylphosphatidylinositol (GPI)-anchors biosynthesis function was predominantly enriched in the dry socket group. This function was critical for host cell membrane processes, such as signal transduction,which may contribute to inflammation and delayed tissue repair [44]. Conversely, the healthy group showed significant enrichment in extracellular matrix (ECM) and cell adhesion molecules (CAMs) pathways, which facilitate cell adhesion and positively impact tissue repair and regeneration [45]. Furthermore, flavonoid and flavonol biosynthesis was highly abundant in the extraction sockets of the healthy group, underscoring their anti-inflammatory and antioxidant effects [46]. These findings highlight the inherent anti-inflammatory properties of the healthy group oral microbiome before extraction, supporting oral health through the production of protective compounds. During the med stage, the bacteria chemotactic pathway was particularly enriched in the dry socket group, potentially facilitating microbial movement to favorable survival sites [47], promoting biofilm formation and exacerbating inflammation. The neuroactive ligand-receptor interaction pathway, also enriched in the dry socket group, may have contributed to heightened pain sensitivity [48]. In contrast, the healthy group exhibited enrichment in the biosynthesis of betaine and indole alkaloids, which support neural regulation and inflammation mitigation [49]. In the post stage, C5-branched dibasic acid metabolism emerged as the most enriched pathway in both groups (healthy and dry socket patients), suggesting a universal metabolic adaptability of the microbial communities. During this phase, these microbes likely played a role in supporting wound healing and responding to inflammation. Notably, the healthy group exhibited enrichment in functions associated with amyotrophic lateral sclerosis [50], suggesting that the microbial community actively supported self-repair and the health of mucosal and neural cells. However, this function appeared disrupted in the dry socket group. In summary, the microbial functions in both the dry socket and healthy groups contributed to tissue recovery during the post-extraction period, with their functions eventually converging. Interestingly, some microbes associated with oral diseases were enriched in the healthy group, while microbes typical of a healthy oral environment were enriched in the dry socket group, possibly due to microbial interactions. These findings prompt further exploration of the connections between symptoms and the oral microbial network.

The oral microbiome network in dry socket patients was distinct from other conditions, and post-operative interventions influenced the structure of the microbial network. Analysis revealed that the interactions within the dry socket group were stronger and more closely connected than in the healthy group, both before and after extraction, suggesting that dry socket patients may have been in an unhealthy state prior to extraction [51]. The tightly connected network of the dry socket group may indicate compromised immunity and reduced resilience in restoring microbial balance [52]. Additionally, enhanced material and information transfer efficiency within this network could accelerate the development of dry socket symptoms [53]. Surgical tooth extraction had minimal impact on the healthy group’s microbial network but altered the dry socket group’s network structure. This led to a temporary decrease in nodes, edges, connectivity and edge density during the med stage, along with changes in the linkage ratio of key negative correlation edges. The number of key edges in the post stage was lower than in the med stage, likely due to post-extraction interventions, though the microbial network recovered quickly [54]. Given that microbial networks are interconnected, we hypothesized that the oral microbiota prior to tooth extraction may be related to the occurrence of dry socket after tooth extraction, which we subsequently verified by random forest analysis.

We found that dry socket occurrence could be partially predicted based on oral microbiota using random forest analysis. Initially, we assessed different sampling sites and found that the prediction accuracy of saliva was higher. We then conducted random forest analysis on various sites within the dry socket and healthy groups, confirming that the accuracy of the saliva site remained higher. Finally, we performed a random forest analysis on healthy and dry socket groups in saliva before tooth extraction. The prediction accuracy of the healthy group was 75%. Based on the importance of the prediction accuracy, 20 microbes were screened out, and it was found that there was a large overlap with the microbes obtained from the previous analyses, such as *Haemophilus* and *Fusobacterium*, which also verified the high reliability of the microbes we screened. Although our results establish a link between pre-extraction microbes and dry socket occurrence, the evidence is not strong enough to predict dry socket directly based on these microbes.

Overall, research on the association between oral microbiota and the oral disease dry socket was limited. This study provided valuable insights to support future investigations into this relationship. It explored the temporal dynamics of oral microbiota during different stages of tooth extraction and analyzed microbial changes over time. By employing random forest models, we demonstrated the potential to predict dry socket occurrence based on oral microbiota, providing a promising framework for future studies. However, the study had several limitations. The dataset and sample size were small, and all samples were collected exclusively from China, which may limit the generalizability of the findings. Larger cohorts in future studies could yield more representative and reliable results. Additionally, the sequencing depth was limited to the amplicon level, potentially missing significant microbes that could play a role in dry socket development. Furthermore, the preliminary findings require validation through additional experiments, and further research is needed to uncover the mechanisms by which key microbes might influence dry socket development.

## Conclusion

In short, this study examined the oral microbiota of individuals with dry socket, identifying distinct microbial differences between the dry socket and healthy groups throughout the tooth extraction process. The dry socket group exhibited an enrichment of tissue-damaging and inflammation-promoting microbes, notably their oral microbiota demonstrating a diseased state prior to extraction, thereby predisposing them to this condition. Key microbial biomarkers were identified, offering valuable insights for future research and the development of potential diagnostic tools. The surgical extraction process was found to impact oral immunity and microbiota interactions, while postoperative interventions played a critical role in stabilizing the microbial environment, supporting beneficial microbes and promoting oral health. Additionally, we proposed a preliminary framework for predicting the risk of dry socket before extraction, paving the way for preventative strategies.

## Supplementary Material

Supplementary_Figure_4.pdf

Supplementary_Figure_3.pdf

Supplementary_Figure_5.pdf

Graphic ab r.pdf

Supplementary_Figure_1.pdf

Supplementary_Figure_2.pdf

Supplementary_Tables.xlsx

## Data Availability

The raw sequence data reported in this paper have been deposited in the Genome Sequence Archive in the National Genomics Data Center, China National Center for Bioinformation/Beijing Institute of Genomics, Chinese Academy of Sciences (GSA: CRA8922) that are publicly accessible at https://ngdc.cncb.ac.cn/gsa. Project number is PRJCA9613. The data and scripts used are saved in GitHub https://github.com/lyuhujie/HujieLyu-DrySocket_OralMicrobiota

## Notes

The study was approved by the Ethics Committee of Peking University Hospital of Stomatology Biomedical (Approval Number: PKUSSIRB - 2,024,100,086), and written informed consent was obtained from all participants. Authors deny any conflicts of interest.
