# Supplementary material for: Correlation between oral microbiota and dry socket at different time periods on tooth extraction
Source: J Oral Microbiol. 2025 Apr 4;17(1):2485210. doi: 10.1080/20002297.2025.2485210 (PMC11980198; doi:10.1080/20002297.2025.2485210)
Supplement: Supplementary_Figure_4.pdf [file ZJOM_A_2485210_SM6570.pdf]

# Supplementary Figure 4.1

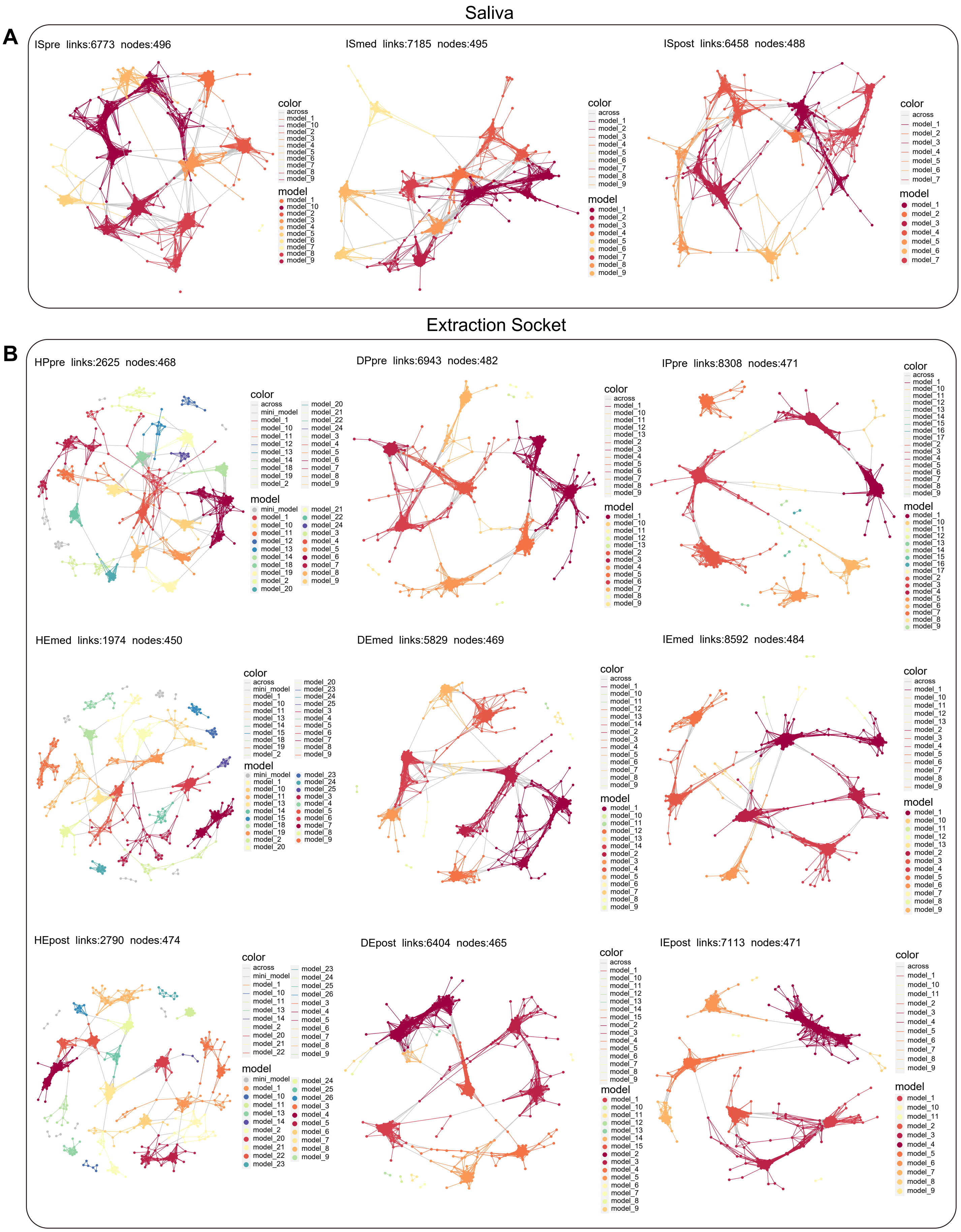

Supplementary figure 4.1
A. Microbial network diagram of saliva microbes in the impaired group at different sampling times: pre, med, and post of the sampling time stages.
B. Comparison of oral microbial networks at different sampling times among the health group, dry socket group, and impaired group in the extraction socket. In all figures, the top left corner indicates the sample name, total number of edges connecting nodes, and the number of nodes. The legend on the right shows the colors corresponding to the edges and nodes.
In the sample names, H represents Health, D represents Dry socket, and I represents Impaired wound; S represents Saliva, P represents the pre-extraction sampling site Periodontal pocket, and E represents the post-extraction sampling site Extraction socket; pre, med, and post represent the three different sampling time periods.

# Supplementary Figure 4.2

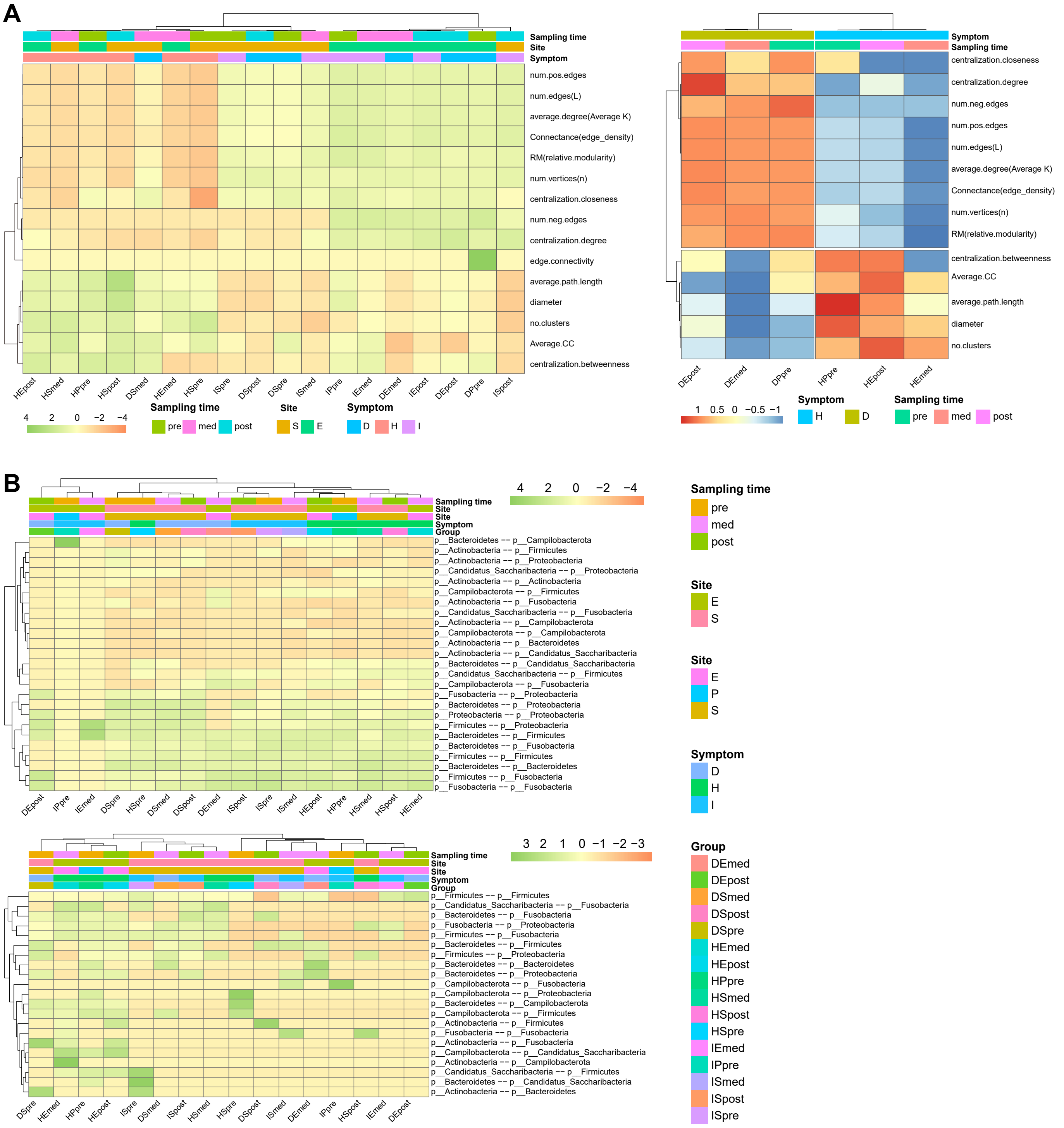

# Supplementary Figure 4.2

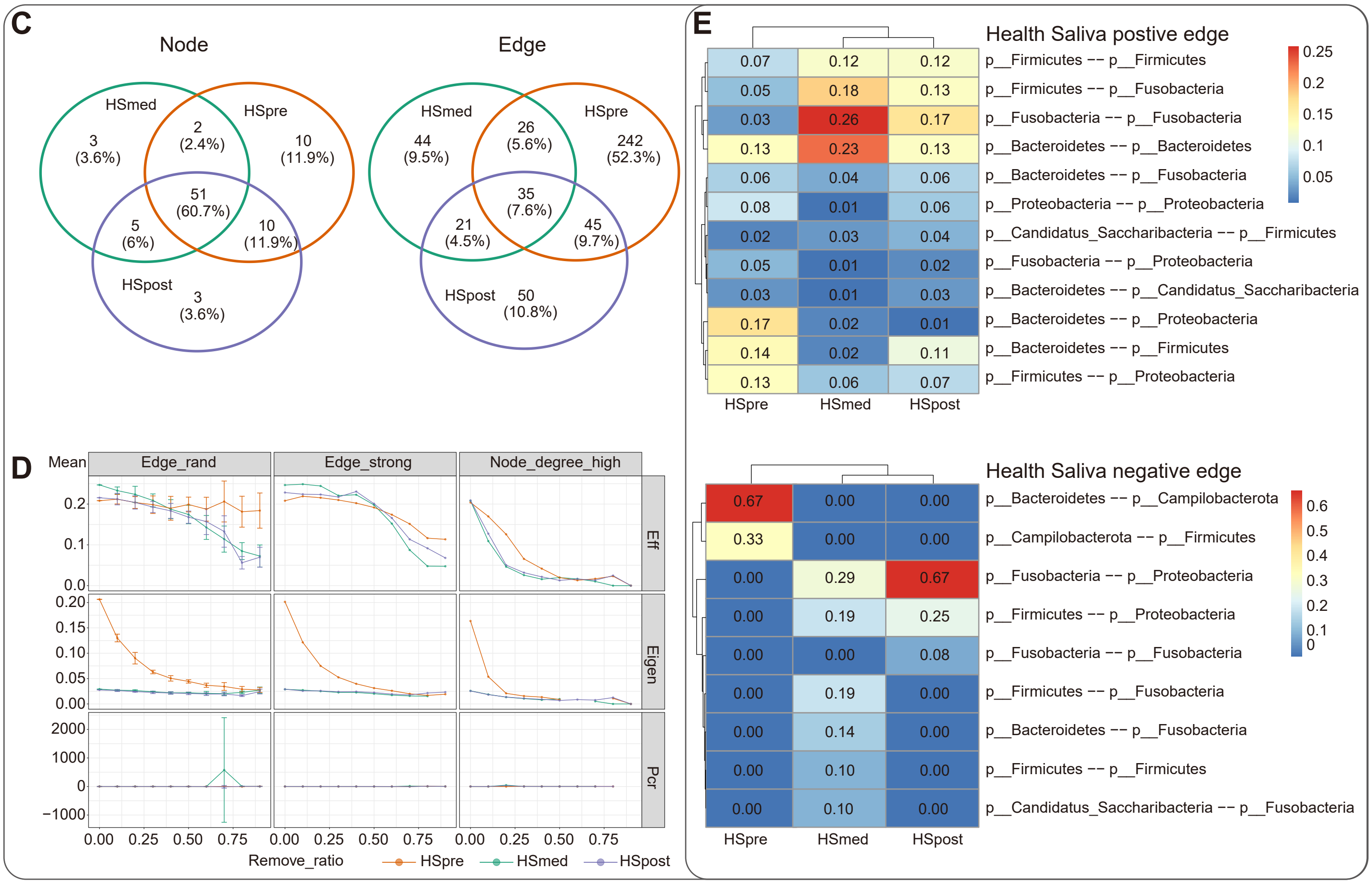


Supplementary figure 4.2

A. Heatmaps of microbial network properties for all samples and individual post-extraction socket samples in the dry socket and health groups at different sampling times. The left figure shows the differences in microbial network properties between the dry socket and health groups, while the right figure shows the differences across all groups. The vertical axis represents the key network properties, with clustering analysis performed on both the horizontal and vertical axes, annotated by symptoms, sampling time, and sampling site.

B. Heatmaps of microbial abundance showing positive and negative correlations in the microbial networks of different groups. In figure A, the upper heatmap represents the abundance of microbes with positive correlations, while the lower heatmap represents those with negative correlations, both with clustering. The annotations are based on sampling time, sampling site, tooth-extraction symptoms, and specific groupings. The greener the color, the more enriched the corresponding positively or negatively correlated microbes are in the respective groups, while the more orange, the less enriched.

C. Venn diagrams of edges and nodes in the microbial networks of saliva from the health group at different periods.

D. Stability analysis of microbial networks in saliva at different periods in the dry socket group. This represents the impact of randomly removing corresponding edges or nodes on network characteristics or performance. Each column from left to right represents randomly removing edges, removing important strong edges, and removing important nodes. Each row from top to bottom represents network efficiency, eigenvector centrality, and pathogen containment ratio.

E. The distribution of the number of phylum-level microbes with positive and negative correlations in the microbial networks of saliva at different periods in the health group. The numbers in the figure indicate the ratio of positive edges to all edges in the network.

## Supplementary Figure 4.3

**A**

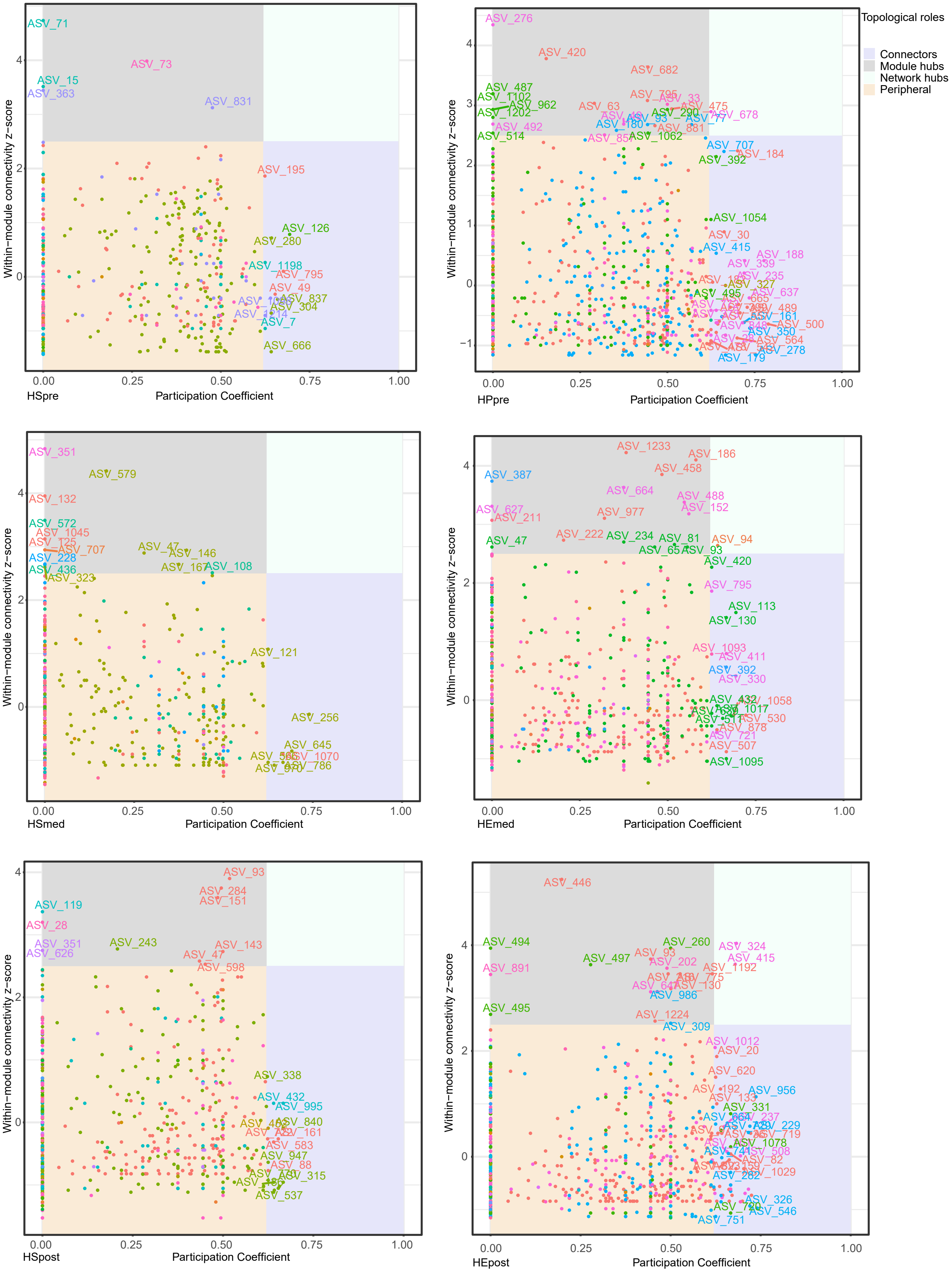

# Supplementary Figure 4.3

**B**

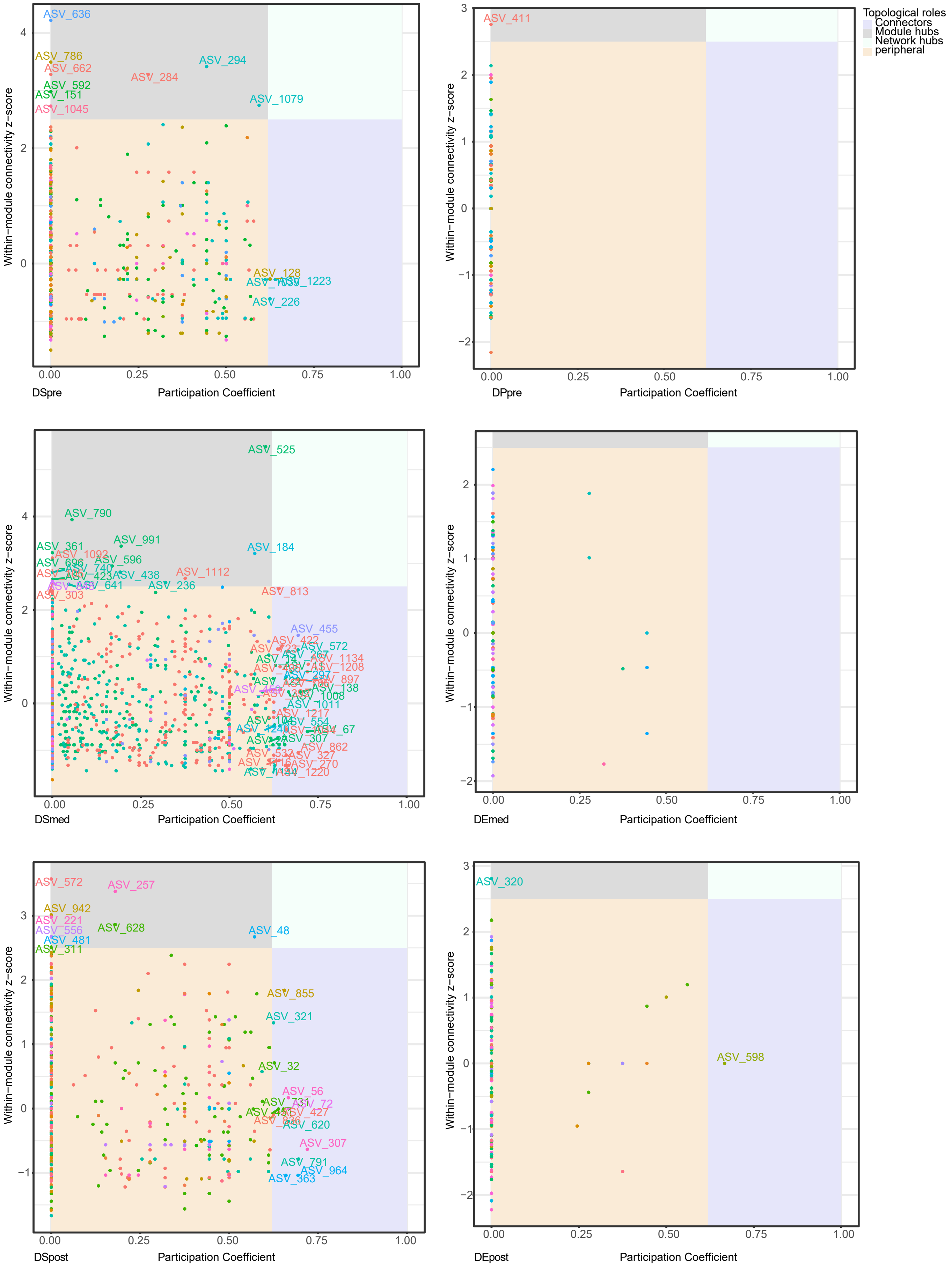

# Supplementary Figure 4.3

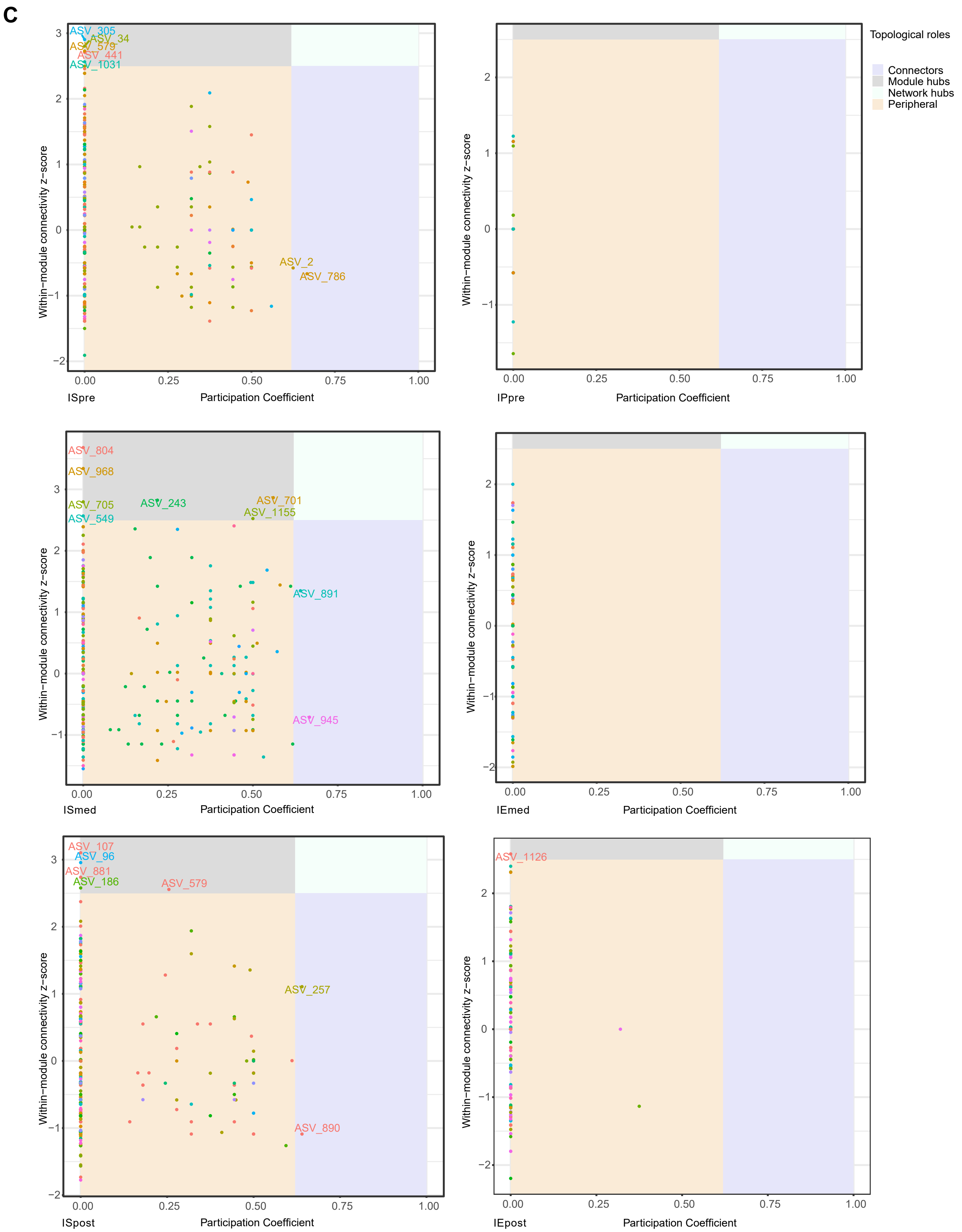

Supplementary figure 4.3
A. ZiPi scatter plots of saliva and extraction socket in the health group during the pre, med and post time stage. Based on the within-module connectivity (Zi) and among-module connectivity (Pi) calculations, and the topological characteristics of nodes, the node attributes can be classified into four types: Module hubs (nodes with high connectivity within a module), Connectors (nodes with high connectivity between two modules), Network hubs (nodes with high connectivity across the entire network), and Peripherals (nodes with low connectivity both within and between modules).
B. ZiPi scatter plots of saliva and extraction socket in the dry socket group during the pre, med and post stage.
C. ZiPi scatter plots of saliva and extraction socket in the impaired healing group during the pre, med and post stage.
In the sample names, H represents Health, D represents Dry socket, and I represents Impaired wound; S represents Saliva, P represents the tooth-extraction sampling site Periodontal pocket, and E represents the post-extraction sampling site Extraction socket; pre, med and post represent the three different sampling time stages.
