# Supplementary material for: Correlation between oral microbiota and dry socket at different time periods on tooth extraction
Source: J Oral Microbiol. 2025 Apr 4;17(1):2485210. doi: 10.1080/20002297.2025.2485210 (PMC11980198; doi:10.1080/20002297.2025.2485210)
Supplement: Supplementary_Figure_3.pdf [file ZJOM_A_2485210_SM6569.pdf]

Supplementary Figure 3.1

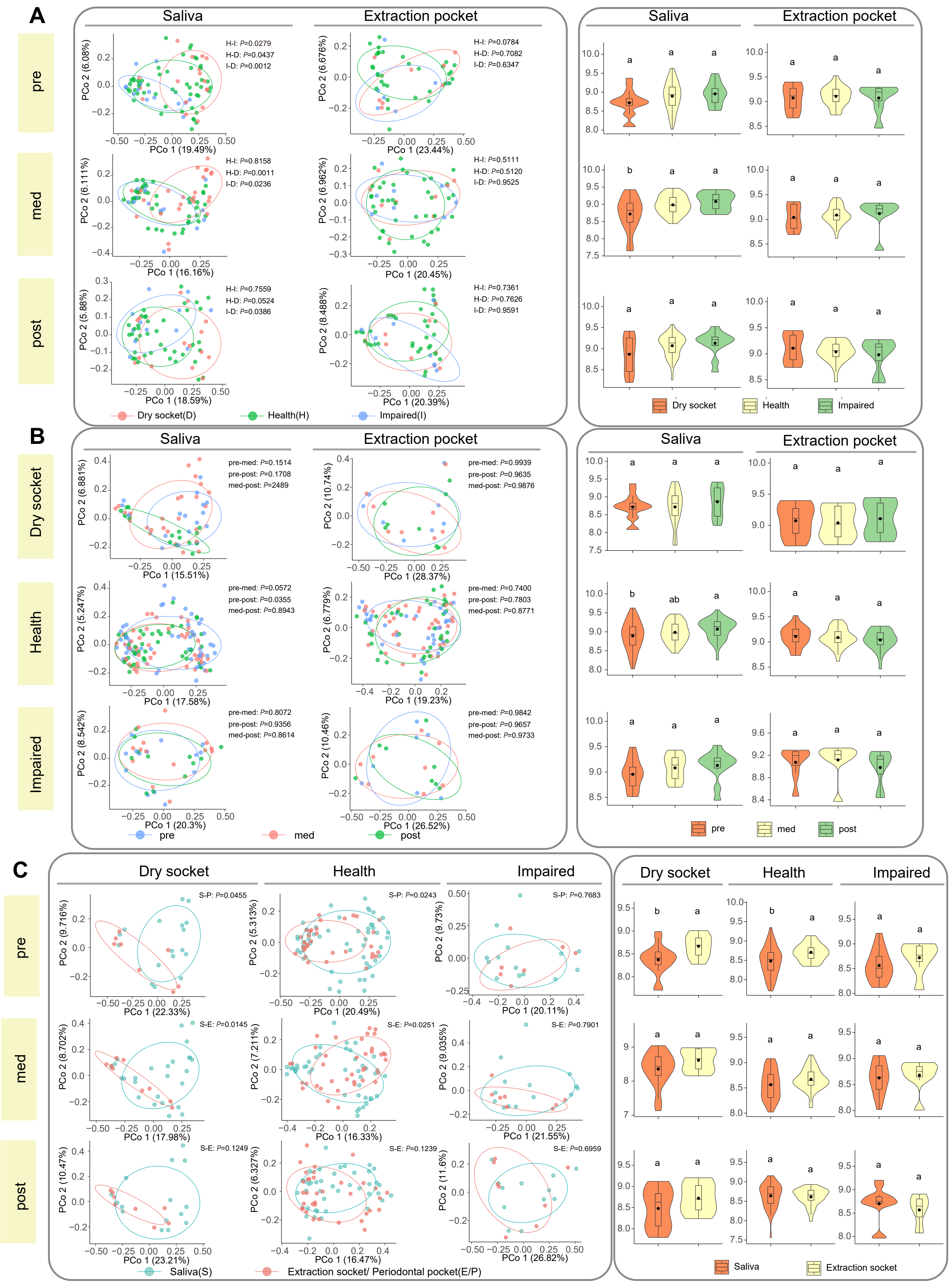

Supplementary Figure 3.1

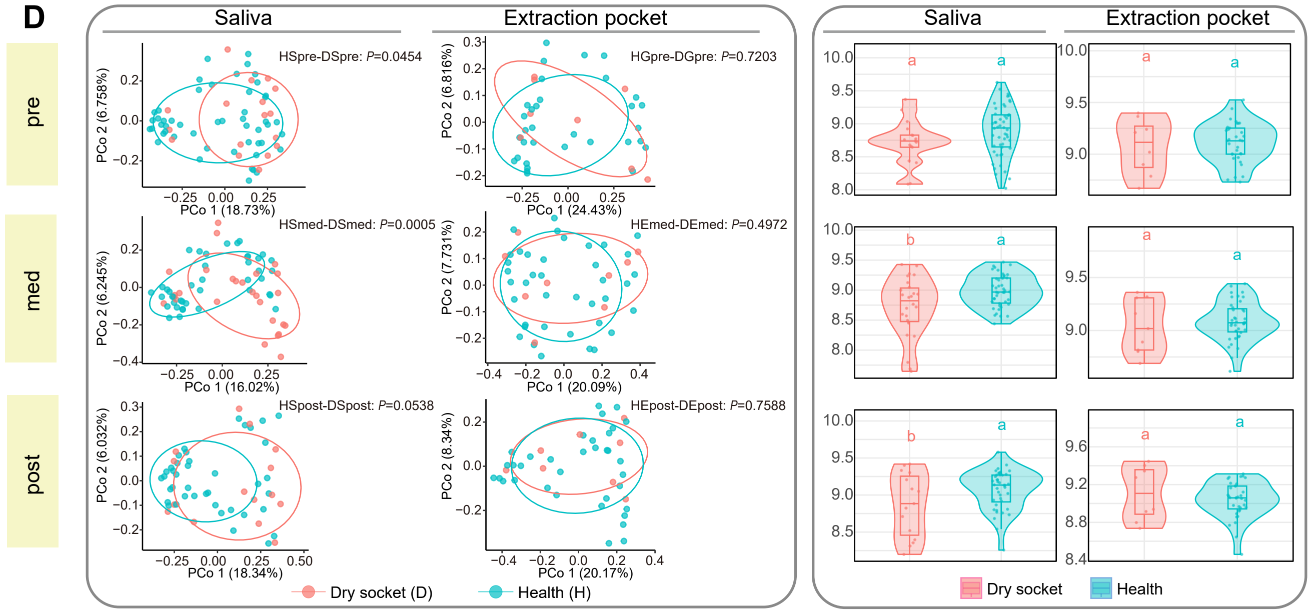

Supplementary figure 3.1
A. Unconstrained PCoA (left figure) (for principal coordinates PCo1 and PCo2) using Bray-Curtis distances analyzes the grouping by different sampling times and sampling sites, and compares the differences in the three symptoms within the corresponding groups ($P < 0.05$ indicates statistical significance, analyzed by Adonis' permutational multivariate analysis of variance (PERMANOVA)). The Chao1 index is used to assess microbial community diversity. The right figure of A groups by different sampling times and sampling sites and compares the Chao1 index among the three symptoms within the corresponding groups. The horizontal lines within the boxes represent the median. The tops and bottoms of the boxes represent the 75th and 25th percentiles, respectively. The upper and lower whiskers extend to data no more than 1.5 times the interquartile range from the upper and lower edges of the box. The overall shape of the violin plot helps identify data skewness and multimodality.
B. Classified by different sampling sites and symptoms, the left figure studies the oral microbial beta diversity, and the right figure studies the alpha diversity across three time periods.
C. Classified by different symptoms and different sampling times, the left figure studies the oral microbial beta diversity, and the right figure studies the alpha diversity across three time periods. In the beta diversity plots, different colors represent different groups, with each point representing a sample. In the alpha diversity plots, the letters above each graph indicate whether there are significant differences between groups ($P < 0.05$), with different letters indicating differences.
D. Divide into six groups based on different sampling times and sampling sites to compare the differences in beta diversity of microbes between health and dry socket patients in the corresponding groups, as well as the alpha diversity according to the Chao1 index.
In the sample names, H represents Health, D represents Dry socket; S represents Saliva, P represents the pre-extraction sampling site Periodontal pocket, and E represents the post-extraction sampling site Extraction socket; pre, med, and post represent the three different sampling time stages.

Supplementary Figure 3.2

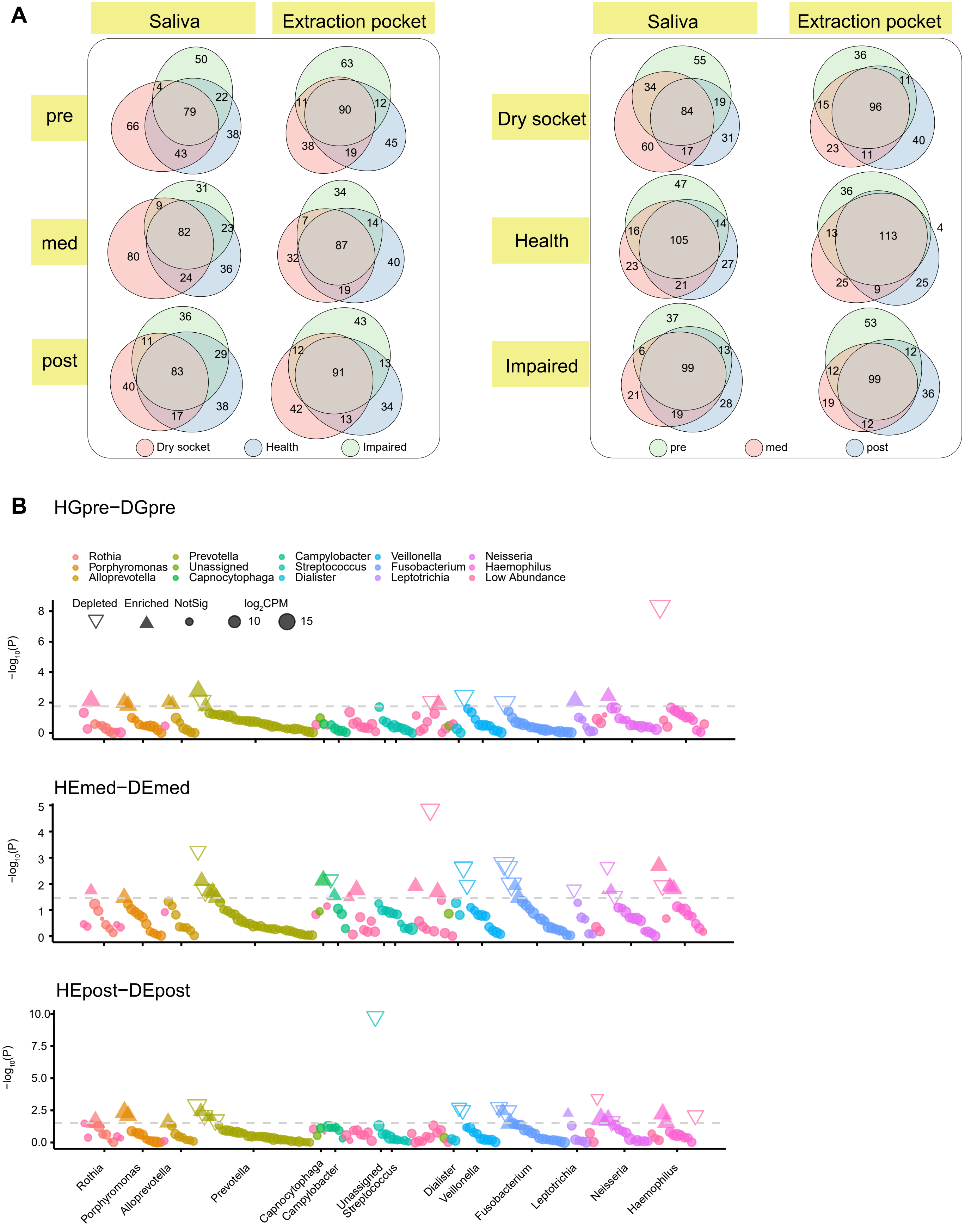

Supplementary figure 3.2

A. Venn diagrams can simplify complex relationships and visually display the intersections, unions, and complements of sets. The left figure is a Venn diagram comparing the relationships of patients with three symptoms grouped by different sampling sites and times. The right figure is a Venn diagram showing the relationships among oral microbes at three different time stages, grouped by different sampling sites and symptoms.

B. The Manhattan plot shows the enriched OTUs in the post-extraction socket during pre, med, and post stages in dry socket and health groups. Each point or triangle represents an OTU. Enriched OTUs in the dry socket or health groups are represented by hollow or solid triangles, respectively (FDR-adjusted $P < 0.05$, Wilcoxon rank-sum test). OTUs are arranged by taxonomy and colored according to the genus level. CPM stands for counts per million.

Supplementary Figure 3.3

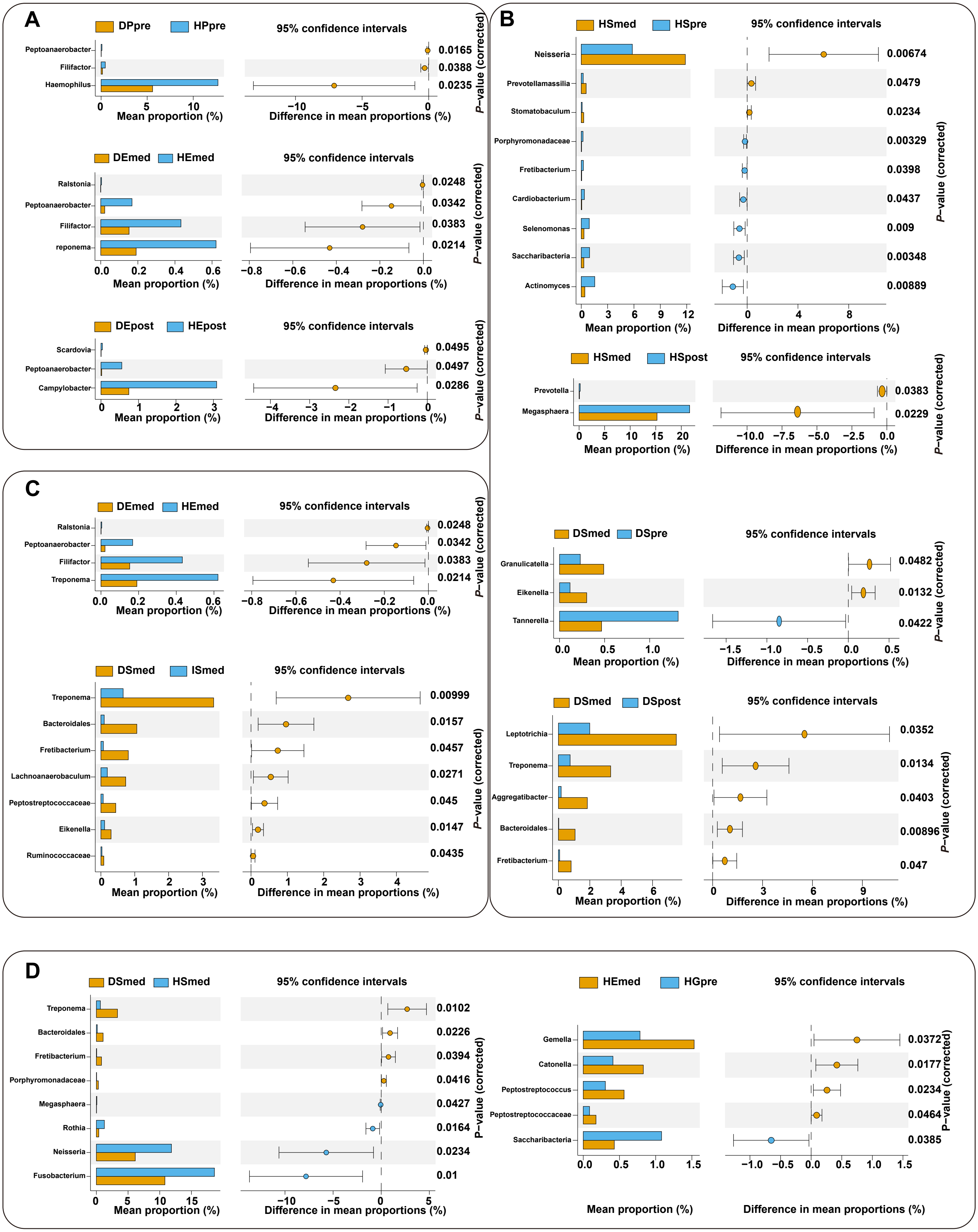

Supplementary figure 3.3

A. Comparison of significant differential microbes in the extraction socket between dry socket and health groups during pre, med, and post stages. Data with $P < 0.05$ are shown within the 95% confidence interval. The expanded parts of the bar charts indicate the magnitude of differences between the groups.

B. Expanded bar charts of microbial differences at different sampling times for the same symptom samples. The top two sets of charts show the differences in microbial counts over different time stages in saliva samples from the health group. The bottom two sets of charts display the differences in microbial counts over different time stages in saliva samples from the dry socket group.

C. The top figure compares the differential microbiota at the tooth extraction site between the dry socket and health groups in the med stage, while the bottom figure compares the salivary microbiota composition differences between the dry socket and impaired groups during the med stage.

D. The left figure compares the differences in saliva microbes between the health and dry socket groups in the med stage.
The right figure compares significant differential microbes in the extraction socket between pre and post stages in the health group. No significant differences were found in the comparisons of the remaining groups.

Supplementary Figure 3.4

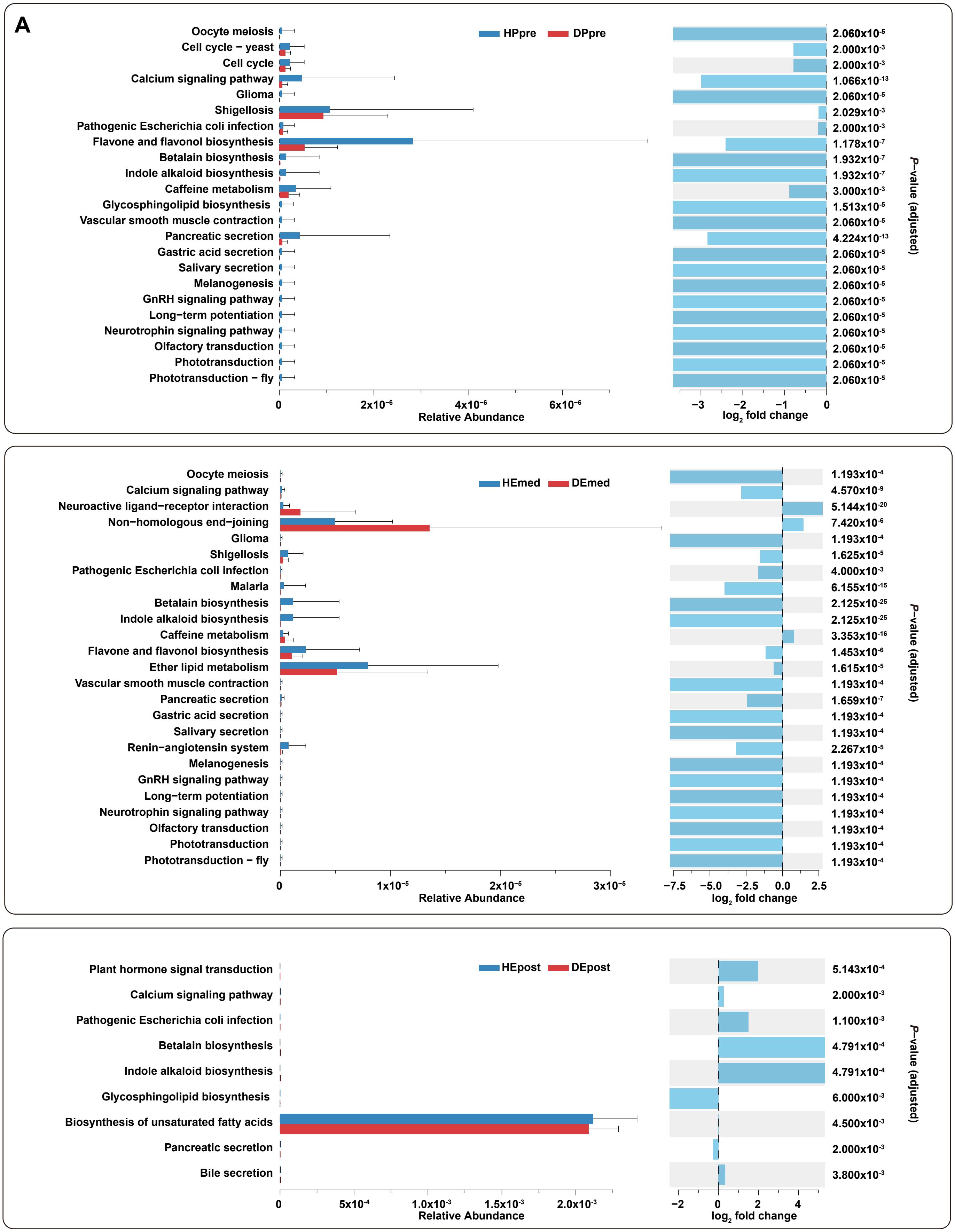

Supplementary Figure 3.4

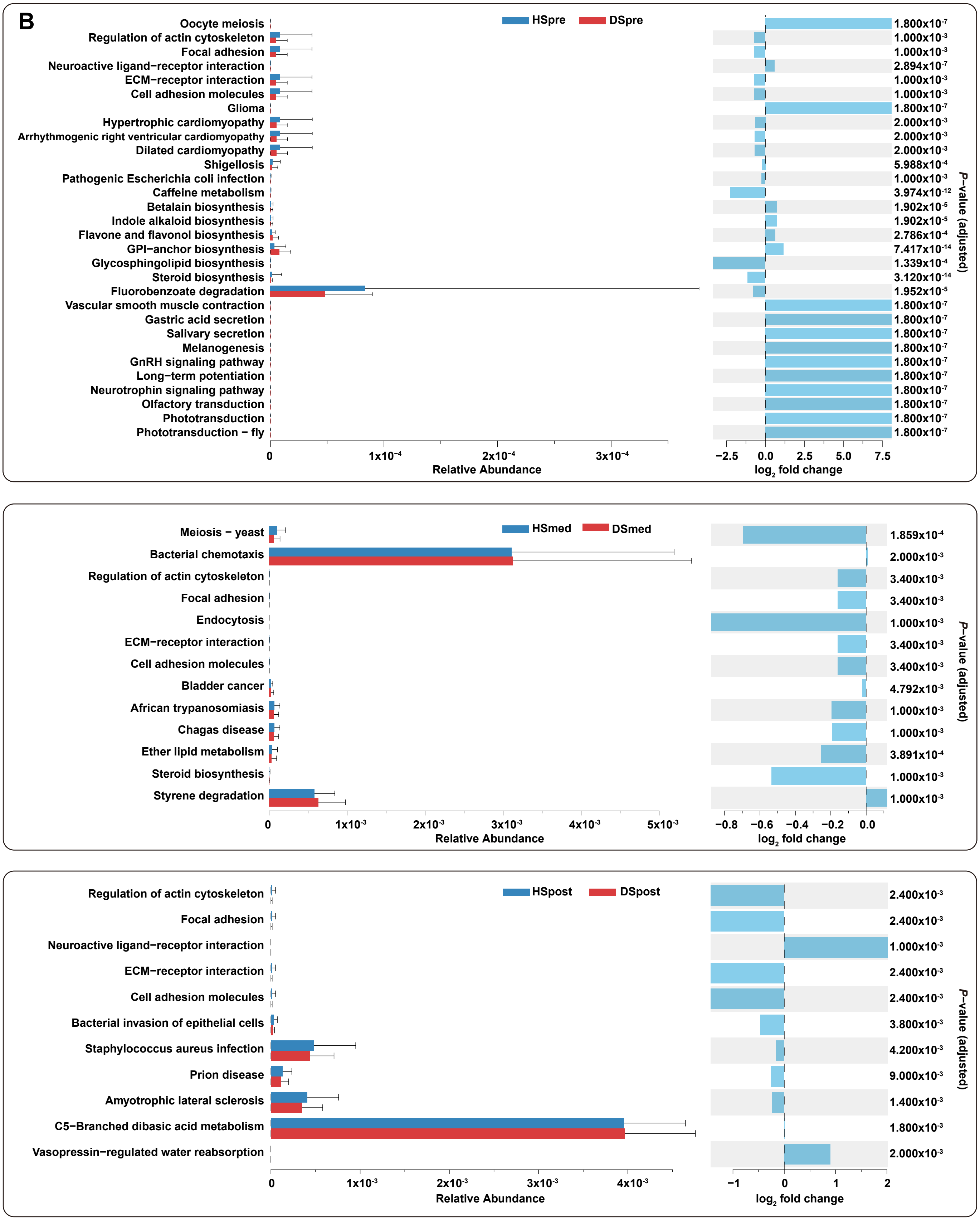

Supplementary figure 3.4
A. Expanded bar charts comparing the relative abundance of significantly different functions of microbes in the extraction socket between the health group and the dry socket group during the pre, med, and post time stage. The top chart shows the pre stage, the middle chart shows the med stage, and the bottom chart shows the post stage.
B. Expanded bar charts comparing the relative abundance of significantly different functions of microbes in saliva between the health group and the dry socket group during the pre, med and post time stages. The top chart shows the pre stage, the middle chart shows the med stage, and the bottom chart shows the post stage.
