# Supplementary material for: Correlation between oral microbiota and dry socket at different time periods on tooth extraction
Source: J Oral Microbiol. 2025 Apr 4;17(1):2485210. doi: 10.1080/20002297.2025.2485210 (PMC11980198; doi:10.1080/20002297.2025.2485210)
Supplement: Supplementary_Figure_5.pdf [file ZJOM_A_2485210_SM6568.pdf]

Supplementary Figure 5

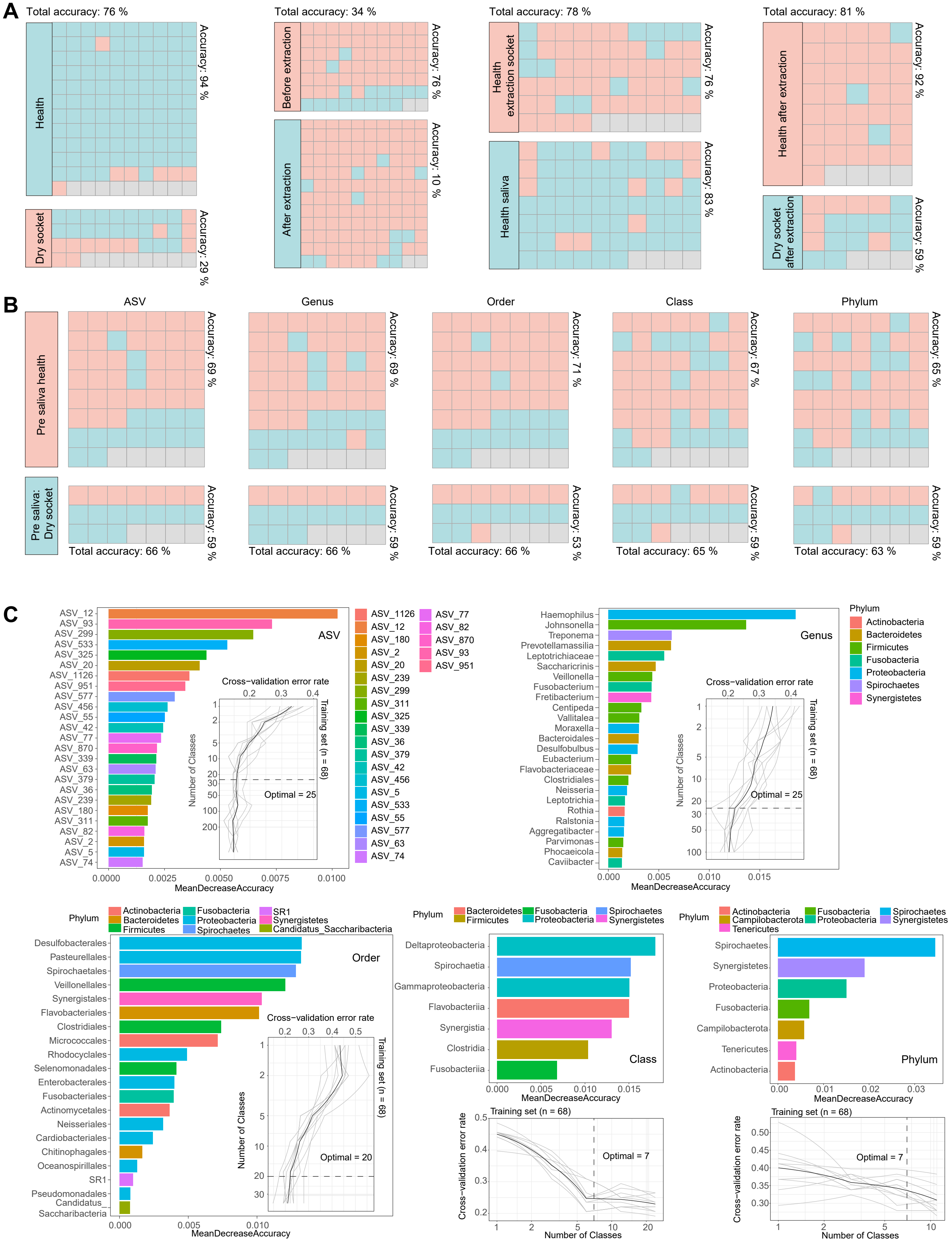

Supplementary Figure 5

Supplementary figure 5
A. The prediction of oral microbiota in different symptoms, sampling times, health group’s tooth-extraction sites, and different symptoms after tooth-extraction using a random forest model. In figure A, the first group on the left shows blue blocks for predictions of health and red blocks for predictions of dry socket. The second group from the left shows red blocks for predictions of pre stage and blue blocks for after extraction stage. The third group from the left shows red blocks for predictions of the extraction socket in the health group and blue blocks for predictions of saliva in the health group. In the last group, red blocks indicate predictions of the symptom after extraction, and blue blocks indicate predictions of the symptom after extractionin. The prediction accuracy for each group is shown on the right, with the overall prediction accuracy at the top.
B. The prediction of dry socket and health groups in the salivary and pre stage using a random forest model. The species taxonomy is evaluated separately to find the taxa with the highest prediction accuracy. From left to right, the accuracy comparisons are shown for ASV level, genus level, order level, class level, and phylum level predictions for dry socket and health groups. Red blocks indicate predictions of the health group for pre stage and salivary, and blue blocks indicate predictions of dry socket for pre-extraction saliva.
C. The bar chart of important feature microbes in the random forest prediction and the line plot of ten-fold cross-validation error. Biomarker taxa are ranked in descending order of importance to the model's accuracy. The ten-fold cross-validation curve is used to distinguish the microbiota in the saliva and extraction socket of the dry socket group based on importance and to determine the optimal number of input microbes. The color groups in the bar chart are differentiated by corresponding phylum-level taxa, and the vertical axis of all figures represents the microbes at the respective taxonomic levels.
