# Supplementary material for: Correlation between oral microbiota and dry socket at different time periods on tooth extraction
Source: J Oral Microbiol. 2025 Apr 4;17(1):2485210. doi: 10.1080/20002297.2025.2485210 (PMC11980198; doi:10.1080/20002297.2025.2485210)
Supplement: Graphic ab r.pdf [file ZJOM_A_2485210_SM6567.pdf]

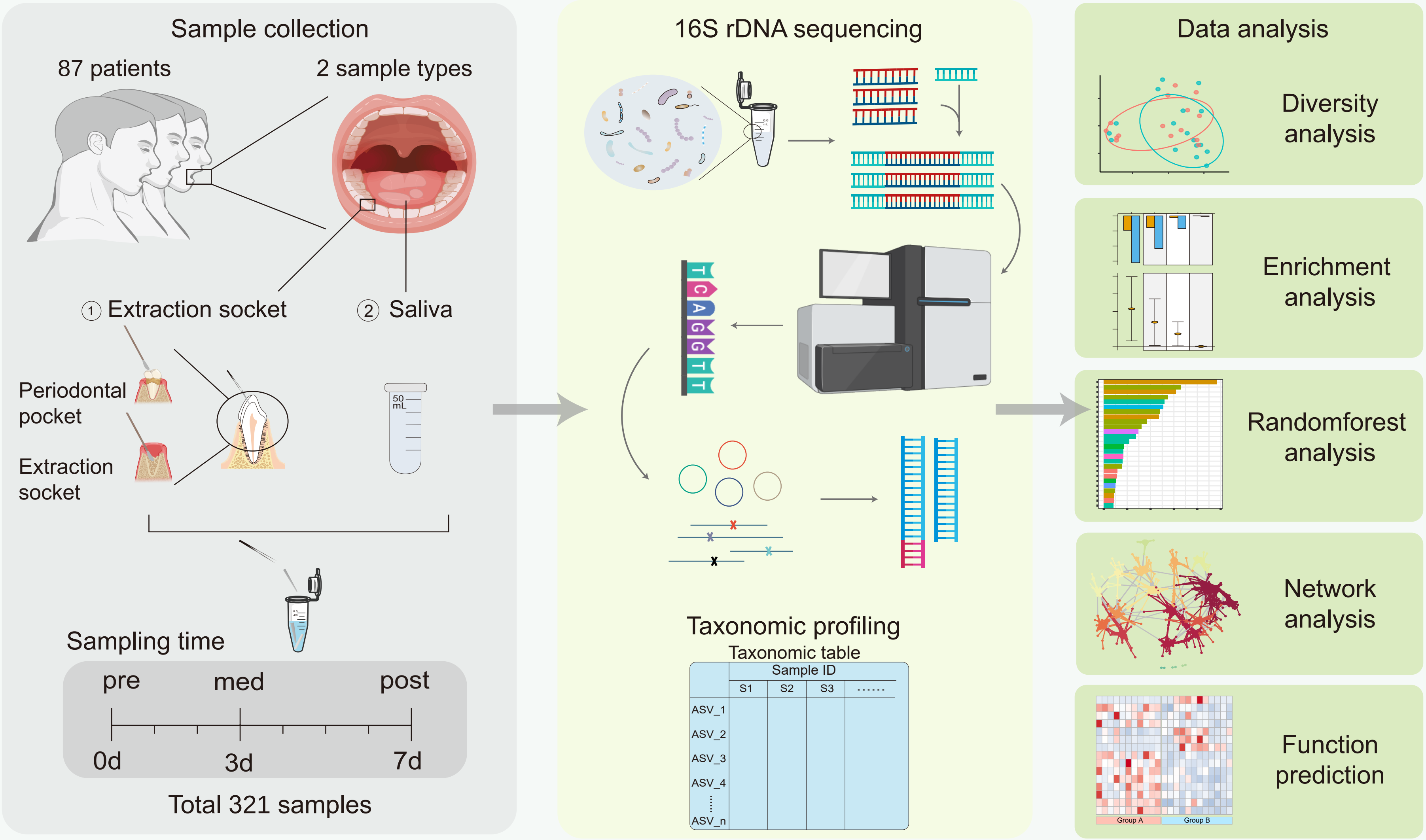
Sample collection
87 patients
2 sample types
① Extraction socket
② Saliva
Periodontal pocket
Extraction socket
50 mL
Sampling time
pre
med
post
0d
3d
7d
Total 321 samples
16S rDNA sequencing
T C A G G T T
Taxonomic profiling
Taxonomic table
Sample ID
S1
S2
S3
......
ASV_1
ASV_2
ASV_3
ASV_4
ASV_n
Data analysis
Diversity analysis
Enrichment analysis
Randomforest analysis
Network analysis
Function prediction
Group A
Group B

Graphic abstract description:

A total of 321 samples from 87 individuals were used to investigate the association between oral microbiota of healthy and dry socket group. Through amplicon sequencing analysis, it was found that the microbial network in the dry socket group was significantly different from healthy group. Moreover, the microbial diversity and composition in the dry socket group at different sampling time also differed markedly from healthy group. Changes in microbial composition were observed to influence microbial functions, which might be the underlying cause of dry socket. We hope to achieve early prediction of dry socket by investigating microbial changes in the pre-extraction stage.
