# Supplementary material for: Correlation between oral microbiota and dry socket at different time periods on tooth extraction
Source: J Oral Microbiol. 2025 Apr 4;17(1):2485210. doi: 10.1080/20002297.2025.2485210 (PMC11980198; doi:10.1080/20002297.2025.2485210)
Supplement: Supplementary_Figure_1.pdf [file ZJOM_A_2485210_SM6566.pdf]

Supplementary Figure 1

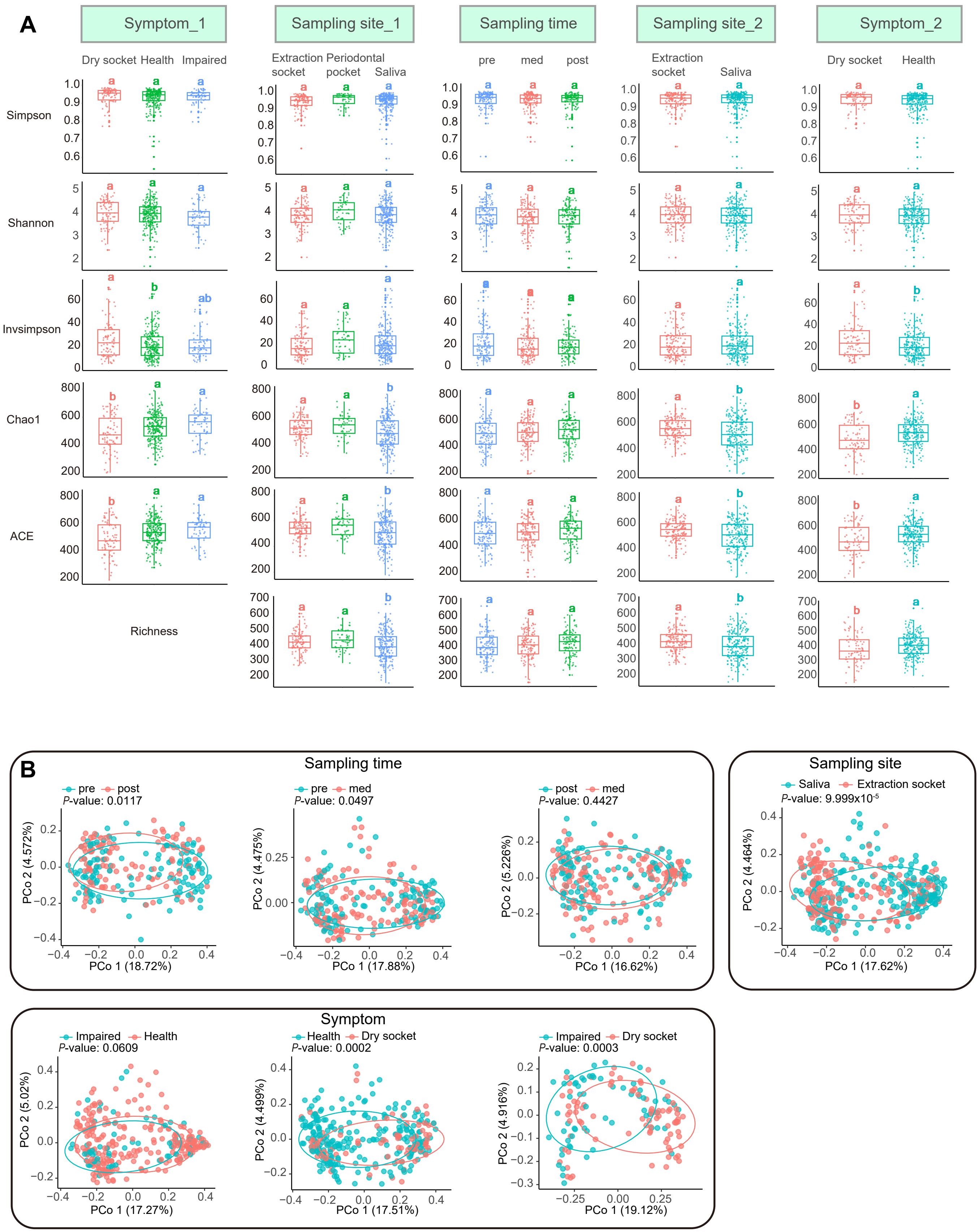

Supplementary Figure 1

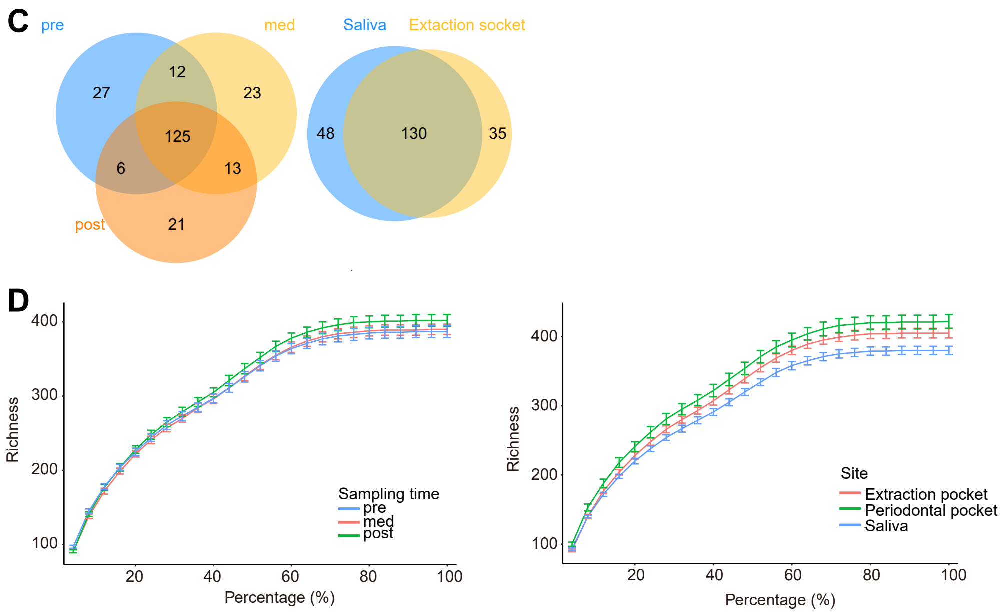

Supplementary Figure 1
A. The alpha diversity of different symptoms, sampling sites, and sampling times, and a comparison of the differences in alpha diversity indices, including Simpson, Shannon, invsimpson, Chao1, and ACE. The horizontal lines within the boxes represent the median. The tops and bottoms of the boxes represent the 75th and 25th percentiles, respectively. The upper and lower whiskers extend to data no more than 1.5 times the interquartile range from the upper and lower edges of the box.
B. Unconstrained PCoA (for principal coordinates PCo1 and PCo2) using Bray-Curtis distances compares the beta diversity differences among different sampling times, sampling sites, and symptoms ($P < 0.05$ is defined as statistically significant, analyzed by Adonis' permutational multivariate analysis of variance (PERMANOVA)).
C. The Venn diagram compares unique and shared microbes at different stages before and after tooth extraction (left figure). It also compares the overlapping microbes between saliva and the extraction socket.
D. The left figure compares the species accumulation rarefaction curves across different time stages. The bottom figure compares the rarefaction curves among the extraction socket, saliva, and the pre-extraction periodontal pocket.
