# Supplementary material for: Correlation between oral microbiota and dry socket at different time periods on tooth extraction
Source: J Oral Microbiol. 2025 Apr 4;17(1):2485210. doi: 10.1080/20002297.2025.2485210 (PMC11980198; doi:10.1080/20002297.2025.2485210)
Supplement: Supplementary_Figure_2.pdf [file ZJOM_A_2485210_SM6565.pdf]

Supplementary Figure 2.1

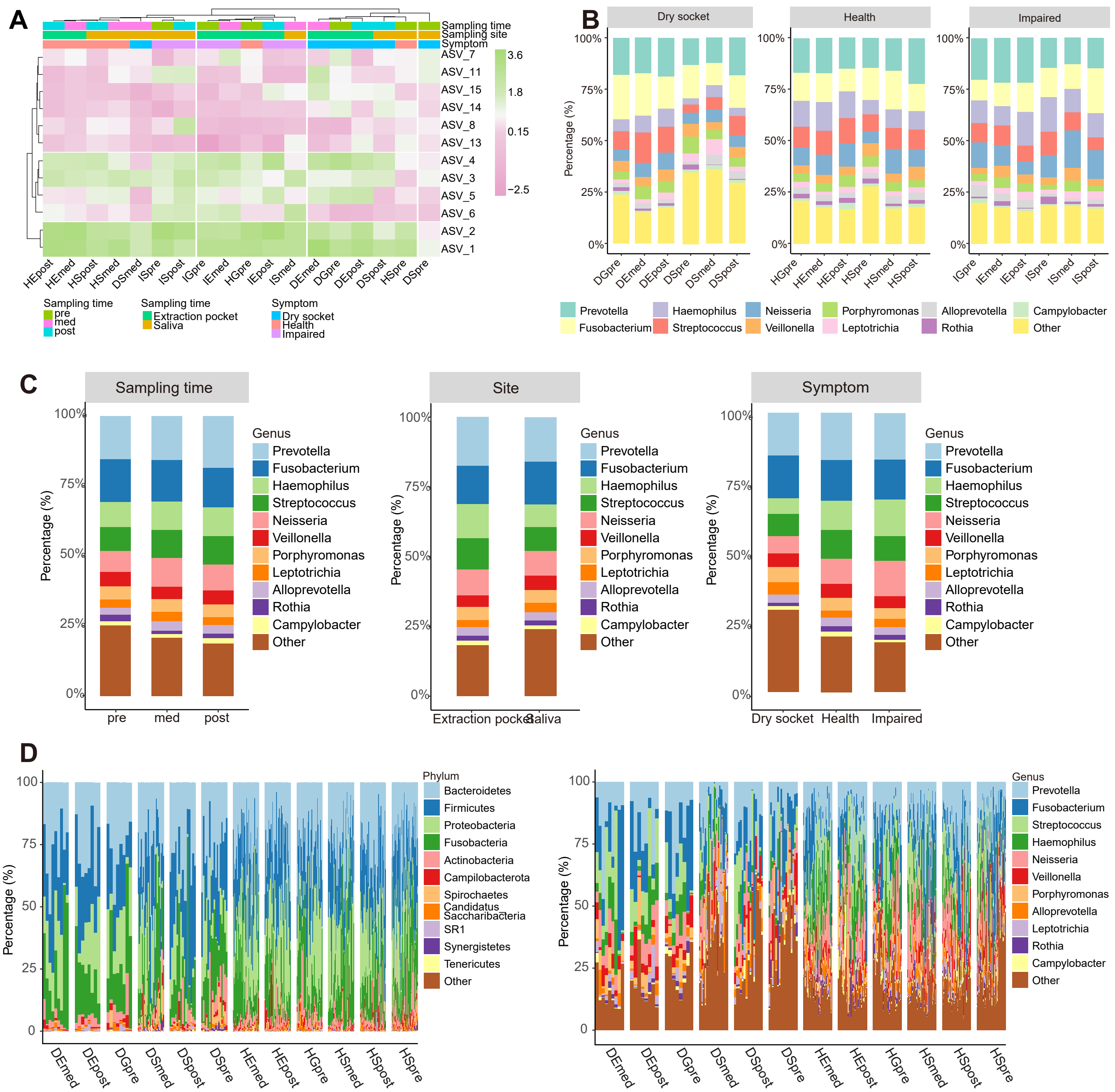

Supplementary figure 2.1

A. The clustering heatmap shows the abundance of microbes under different groupings. The top of figure A displays clusters grouped by sampling time, sampling site, and symptoms, with different colors representing different groups.

B. The stacked bar chart displays the percentage of microbes for all groups, faceted by the three symptoms, and divides the 18 groups into three sections for display.

C. The bar chart of microbial species composition is plotted based on the overall sampling time, sampling site, and symptoms. For Sampling time, the grouping is only by pre, med, and post without distinguishing other groupings. The same applies to the other two grouping methods. The species composition is presented at the genus level.

D. Group comparison of different sampling sites and time between the dry socket and health groups, along with a comparison of microbial composition differences in each sample. The left figure shows the comparison at the phylum level, while the right figure shows the comparison at the genus level.

In the sample names, H represents Health, D represents Dry socket, and I represents Impaired wound; S represents Saliva, P represents the pre-extraction sampling site periodontal pocket, and E represents the post-extraction sampling site Extraction socket; pre, med, and post represent the three different sampling time stages.

Supplementary Figure 2.2

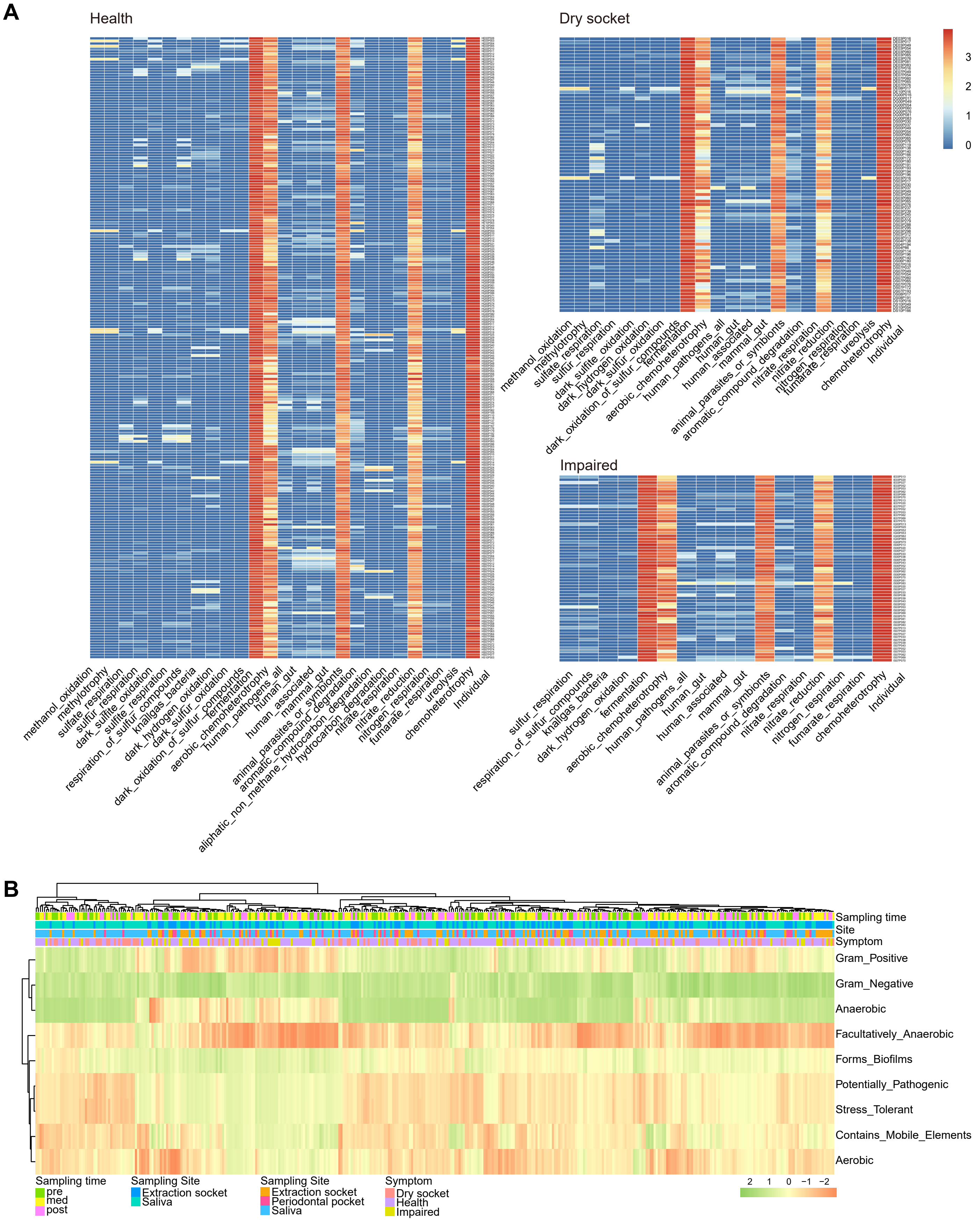

Supplementary figure 2.2

A. Perform functional prediction for all samples and group them by health, dry socket, and impaired. The horizontal axis represents the predicted functions for each group, while the vertical axis represents individual sample names. The redder the color, the more enriched the function. Functional prediction analysis is performed using FAPROTAX 1.1.

B. The cluster heatmap analyzes the functional potential and characteristics of microbial communities predicted by BUGBASE for all samples. The clustering of different sampling times, sites, and symptoms is shown at the top of the heatmap, and the names of the related potential functions and characteristics are displayed on the right side.

Supplementary Figure 2.3

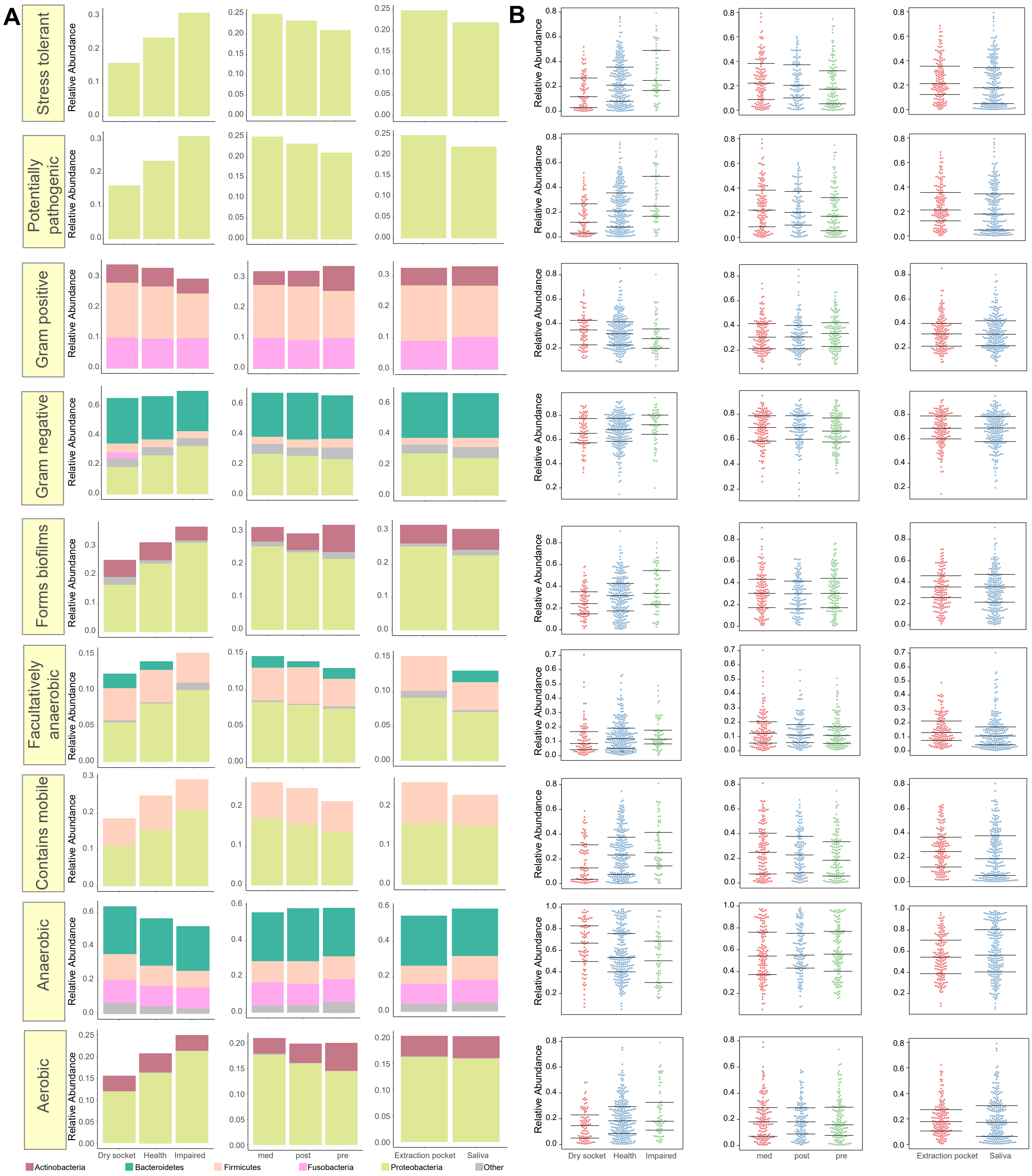

Supplementary Figure 2.3

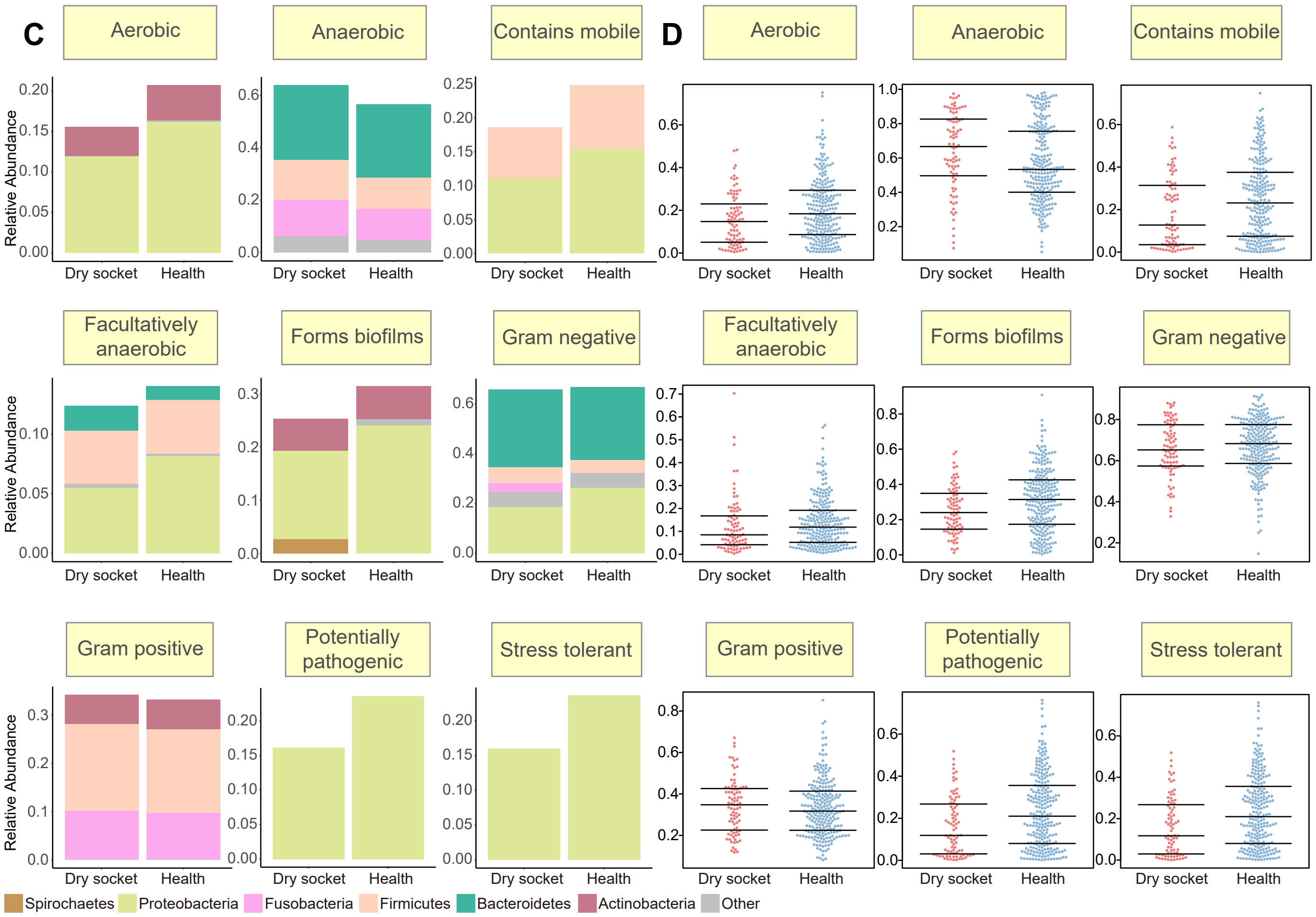

Supplementary figure 2.3

A. Display the relative abundance of microbes that play significant roles in the predicted potential functions and characteristics as stacked bar charts, grouped by different grouping methods. This grouping distinguished impaired group from dry socket and health groups.

B. Display the differences in relative abundance of microbes in various potential functions and characteristics among different groups as scatter plots. The statistical differences between groups in figure B are provided in Supplementary Table 2. This grouping distinguished impaired group from dry socket and health groups.

C, D. Display the relative abundance of microorganisms that play a significant role in the predicted potential functions and traits as stacked bar charts, grouped according to different grouping methods. Additionally, show the differences in the relative abundance of microbes associated with various potential functions and traits in different groups as scatter plots. However, the grouping only compares the health group and the dry socket group, ignoring the impaired group.
